# Detecting ecological traps in human‐altered landscapes: A case study of the thick‐billed longspur nesting in croplands

**DOI:** 10.1002/ece3.9993

**Published:** 2023-04-18

**Authors:** Amber E. Swicegood, Kevin S. Ellison, Marisa Sather, Scott G. Somershoe, Lance B. McNew

**Affiliations:** ^1^ Department of Animal and Range Sciences Montana State University Bozeman Montana USA; ^2^ Northern Great Plains, American Bird Conservancy Bozeman Montana USA; ^3^ Fish and Wildlife Program U.S. Fish and Wildlife Service Glasgow Montana USA; ^4^ Division of Habitat Conservation Land Bird Coordinator U.S. Fish and Wildlife Service Migratory Birds Program Denver Colorado USA

**Keywords:** ecological trap, farmland birds, grassland birds, maladaptive selection, *Rhynchophanes mccownii*, thick‐billed longspur

## Abstract

Conversion of the North American prairies to cropland remains a prominent threat to grassland bird populations. Yet, a few species nest in these vastly modified systems. Thick‐billed longspurs historically nested in recently disturbed or sparsely vegetated patches within native mixed‐grass prairie, but observations of longspurs in spring cereal and pulse crop fields during the breeding season in northeastern Montana, USA, suggest such fields also provide cues for habitat selection. Maladaptive selection for poor‐quality habitat may contribute to ongoing declines in longspur populations, but information on thick‐billed longspur breeding ecology in crop fields is lacking. We hypothesized that crop fields may function as ecological traps; specifically, we expected that crop fields may provide cues for territory selection, but frequent human disturbance would result in reduced reproduction. To address this hypothesis, we compared measures of habitat selection (settlement patterns and trends in abundance) and productivity (nest density, nest survival, and number of young fledged) between crop fields and native grassland sites during 2020–2021. Across both years, settlement patterns were similar between site types and occupancy ranged from 0.52 ± 0.17 SE to 0.99 ± 0.01 on April 7 and 30, respectively. Early season abundance differed by year, and changes in abundance during the breeding season appeared to be associated with precipitation‐driven vegetation conditions rather than habitat type. While an index of nest density was lower in crop than native sites, the number of young fledged per successful nest (2.9 ± 0.18 SE) and nest survival (0.24 ± 0.03 SE; *n* = 222 nests) were similar for crop and native sites. Collectively, the data did not support our ecological trap hypothesis: longspurs did not exhibit a clear preference for crop sites and reproductive output was not significantly reduced. Our results indicate that croplands may provide alternative breeding habitat within a human‐dominated landscape.

## Graphical abstract

Maladaptive selection for poor‐quality habitat may contribute to ongoing declines of some grassland birds, but information on breeding ecology in human‐altered landscapes is lacking. We compared the settlement behavior, seasonal changes in breeding density, and reproductive success of thick‐billed longspurs in native grassland habitats and crop fields in the core of their remaining breeding distribution. We did not find support for our ecological trap hypothesis; preference was similar and precipitation appeared to mediate the effects of habitat type on longspur breeding ecology.

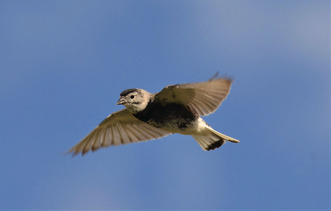

## 1 INTRODUCTION

In North America, grassland birds have experienced steeper long‐term declines than any other avian guild during the past 50 years (Rosenberg et al., 2019; Sauer et al., 2020), and more than 79% of grasslands have been lost since the early 1800s (Samson & Knopf, 1994; White et al., 2000). While factors such as fire suppression, overgrazing, desertification, and the introduction of non‐native plant species have contributed to degradation of native prairies, conversion to cultivated cropland remains one of the greatest threats to grassland ecosystems (Blann, 2006; Ellis et al., 2010; Knapp et al., 1999; Wright & Wimberly, 2013). Indeed, intensification of agricultural practices is considered a leading driver of grassland bird population declines worldwide (Davis et al., 2020; Quinn et al., 2017; Wilson et al., 2005). However, some species do use crop fields for nesting and foraging (Best et al., 1997; Davis et al., 2020). Birds nesting in crop fields face a myriad of hazards: farming operations (e.g., tilling, discing, and harvest) may result in nest destruction (Devries et al., 2008; Santangeli et al., 2018), and soil instability in crop fields may lead to nest failures during floods and heavy rains (Van Pelt et al., 2017). Herbicide and pesticide applications may directly harm adults and nestlings or indirectly affect bird populations by reducing invertebrate food resources (Loss et al., 2015; Pimentel et al., 1995). In addition, most crops grow rapidly into dense monocultures, with vegetation conditions changing from short stature (5–15 cm tall) with large amounts of bare ground to tall (60–70 cm), closed‐canopy conditions within 2–3 months of seeding (Wilson et al., 2005).

Ecological traps occur when there is a mismatch between habitat selection cues and habitat quality (Battin, 2004), and are most commonly identified where human activities produce novel environmental cues or alter habitat quality associated with a particular cue (Hale & Swearer, 2016; Robertson et al., 2013; Simon & Fortin, 2019). An ecological trap differs from a demographic sink in that animals often exhibit strong preference for trap habitat (Gilroy et al., 2011; Pulliam, 1988). Such maladaptive selection leads to negative fitness consequences and reduced population viability (Battin, 2004; Gilroy et al., 2011; Schlaepfer et al., 2002). The ideal free distribution theory that underpins source–sink population models assumes animals exhibit optimal habitat selection when distributing themselves among habitat patches and that the fittest individuals obtain the highest‐quality territories. In reality, individuals likely select habitat according to evolutionarily predisposed cues, and ecological traps are attractive because they provide such cues (Abrams et al., 2012; Delibes et al., 2001; Fletcher Jr et al., 2012; Hale et al., 2015; Hale & Swearer, 2016).

**FIGURE 1:**
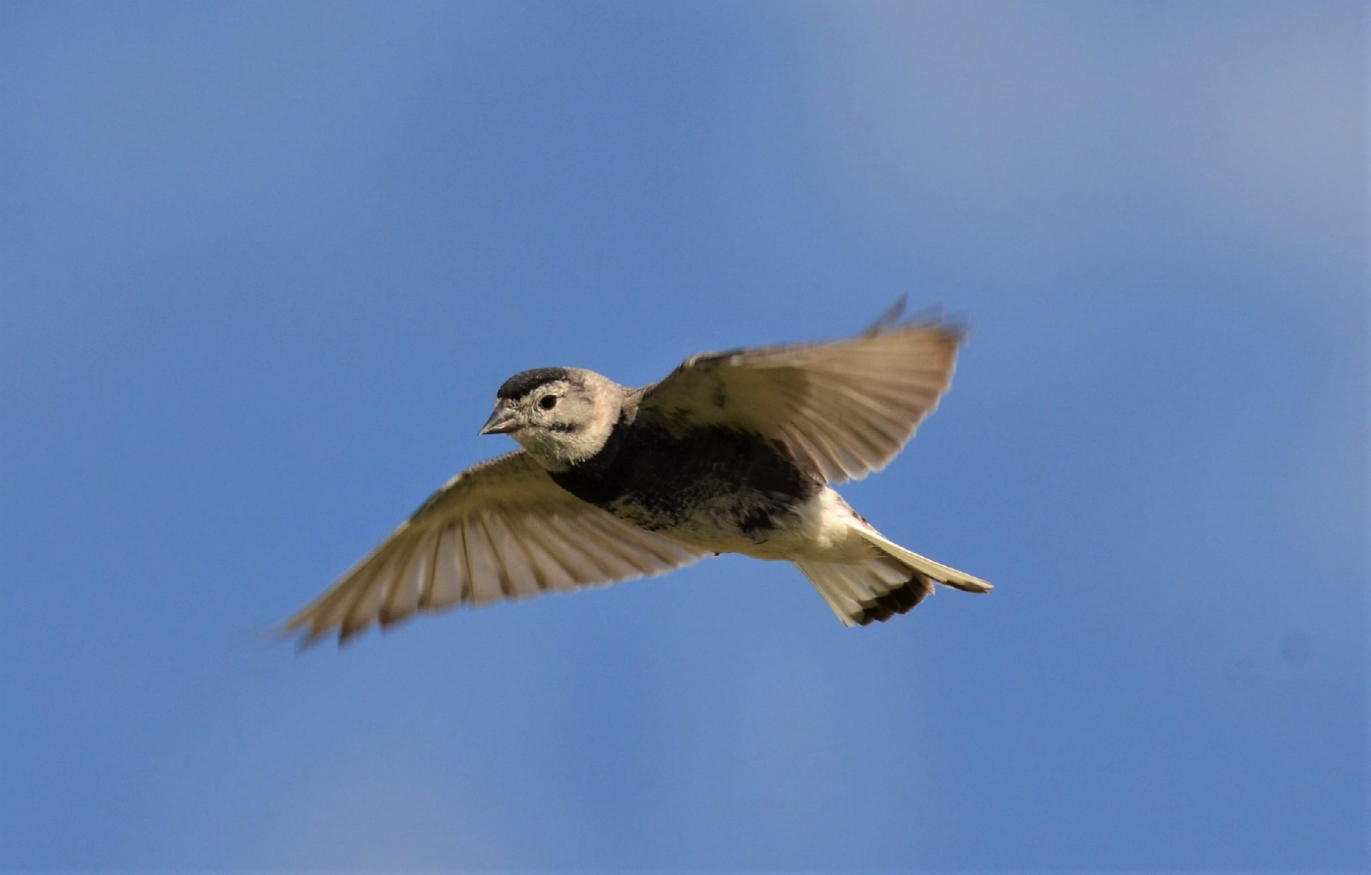
Male thick‐billed longspur (*Rhynchophanes mccownii*) in flight.

The thick‐billed longspur (*Rhynchophanes mccownii*; hereafter “longspur”; Figure 1) is a grassland songbird endemic to the short‐ and mixed‐grass prairies of North America (Knopf, 1996). Like most obligate grassland birds, populations of longspurs have declined precipitously (4% per year on average) since the advent of the North American Breeding Bird Survey (Rosenberg et al., 2019; Sauer et al., 2020), but mechanisms driving the decline are poorly understood. Longspur habitat is patchy within native mixed‐grass prairie, resulting in clustered distributions at the landscape level (i.e., high local densities in some areas yet absent from other superficially similar areas; Greer & Anderson, 1989; Lipsey, 2015). As such, longspur habitat preferences limit distributions at regional scales and make this a focal species for federal conservation efforts (Somershoe, 2018). Unlike many other grassland birds, longspurs have a unique preference for recently disturbed or sparsely vegetated habitats and historically relied on large‐scale disturbance regimes to maintain suitable habitat patches through spatial–temporal interactions of soil, precipitation, fire, and intensive periodic defoliation by native herbivores (e.g., bison [*Bison bison*] and locusts [chiefly *Melanoplus spretus*]) (Felske, 1971; Samson et al., 2004; Shaffer et al., 2019; With, 2021). However, these dynamic processes that once shaped prairie ecosystems are largely absent in today's Northern Great Plains (Fuhlendorf & Engle, 2004; Hovick et al., 2015; Samson & Knopf, 1996).

Cropland, or cultivated land annually seeded to crops, is now the dominant disturbance factor producing extensive bare ground at large scales in the Northern Great Plains. Sporadic reports of longspurs occurring in crop fields during the breeding season (Felske, 1971; Shaffer et al., 2019) suggest that some croplands may resemble suitable nesting habitats as longspurs arrive on the breeding grounds in April. However, the selection of crop fields during territory establishment could contribute to population declines if reproductive success is depressed through (1) destruction of nests by discing, seeding, and herbicide/pesticide application; and (2) abandonment of nests or territories in response to rapid changes in vegetation conditions or disturbance by farming activity. If crop fields provide attractive nesting habitats where longspurs experience low reproductive success, modern cultivated landscapes may be operating as ecological traps for this short‐grass prairie specialist.

Identifying an ecological trap involves demonstrating habitat preference (i.e., disproportionate selection) that results in reduced vital rates (e.g., nest survival). Importantly, high population densities do not necessarily equate to preference for that habitat type (Van Horne, 1983; Vickery et al., 1992), and comparisons of relative selection metrics between habitat types are needed to discern preferences. A demonstrated preference for crop fields coupled with reduced reproductive success relative to native prairie would indicate an ecological trap with significant implications for longspur conservation (Lloyd & Martin, 2005; Robertson & Hutto, 2006). Delayed settlement in crop fields after native sites are occupied, coupled with lower reproductive success, would suggest crop fields simply provide spill‐over habitat, which is characteristic of source–sink populations (Gilroy & Sutherland, 2007).

We conducted a 2‐year field study to evaluate whether cultivated fields, primarily cereal and pulse crops, operate as ecological traps for nesting longspurs within the core of their breeding distribution in northeastern Montana, USA. We compared settlement patterns, temporal trends in abundance, and nest density between crop fields (hereafter “crop sites”) and nearby native prairie (hereafter “native sites”), assuming that earlier settlement and higher use of crop sites indicated that either longspurs preferred crop sites for nesting or that native sites were limited (Robertson & Hutto, 2006). We measured reproductive success in both habitat types to assess the relative quality of crop sites as breeding habitats for longspurs. Our objectives were to compare the following in crop and native sites: (1) settlement patterns of territorial male longspurs; (2) longspur abundance and nest densities, as well as changes in abundance over the breeding season; (3) nest survival and fledging success; and (4) structural changes in vegetation during the breeding season. For our ecological trap hypothesis to be supported, we first predicted that crop sites would become fully occupied by longspurs earlier than native sites, indicating earlier settlement and preference for crop habitat. Second, we predicted that longspurs would occur in higher abundance in crop sites early in the season, but abundance would decline over the growing season as crop height increased because longspurs are known to avoid tall, dense vegetation. We predicted that nest densities would reflect bird abundance and longspurs may abandon nests in crop fields when vegetation height increased. Lastly, we predicted that rates of daily nest survival and fledging success would be lower in crop sites due to hazards from farm machinery and increased exposure to weather and predators.

## 2 MATERIALS AND METHODS

### 2.1 Study area and site selection

Our research was conducted in northern Valley County, Montana, USA, which lies within the core of the remaining breeding distribution of longspurs. The climate in this region is semi‐arid with long, cold winters and short, hot summers producing frequent thunderstorms, hail, and flash floods (Cooper et al., 2001). Average daily temperatures range seasonally from below 0 to 25°C. Annual precipitation averages 25–35 cm and typically comes as rain in late May and early June (Lenard et al., 2006; PRISM, 2022). The region is at about 915 m in elevation. Clay shale is the most abundant substrate, and the landscape is dominated by glacial till (Cooper et al., 2001). Northern Valley County is characterized by large expanses of poor soils unsuitable for cultivation and more productive areas used primarily for cereal and pulse crop production.

**FIGURE 2:**
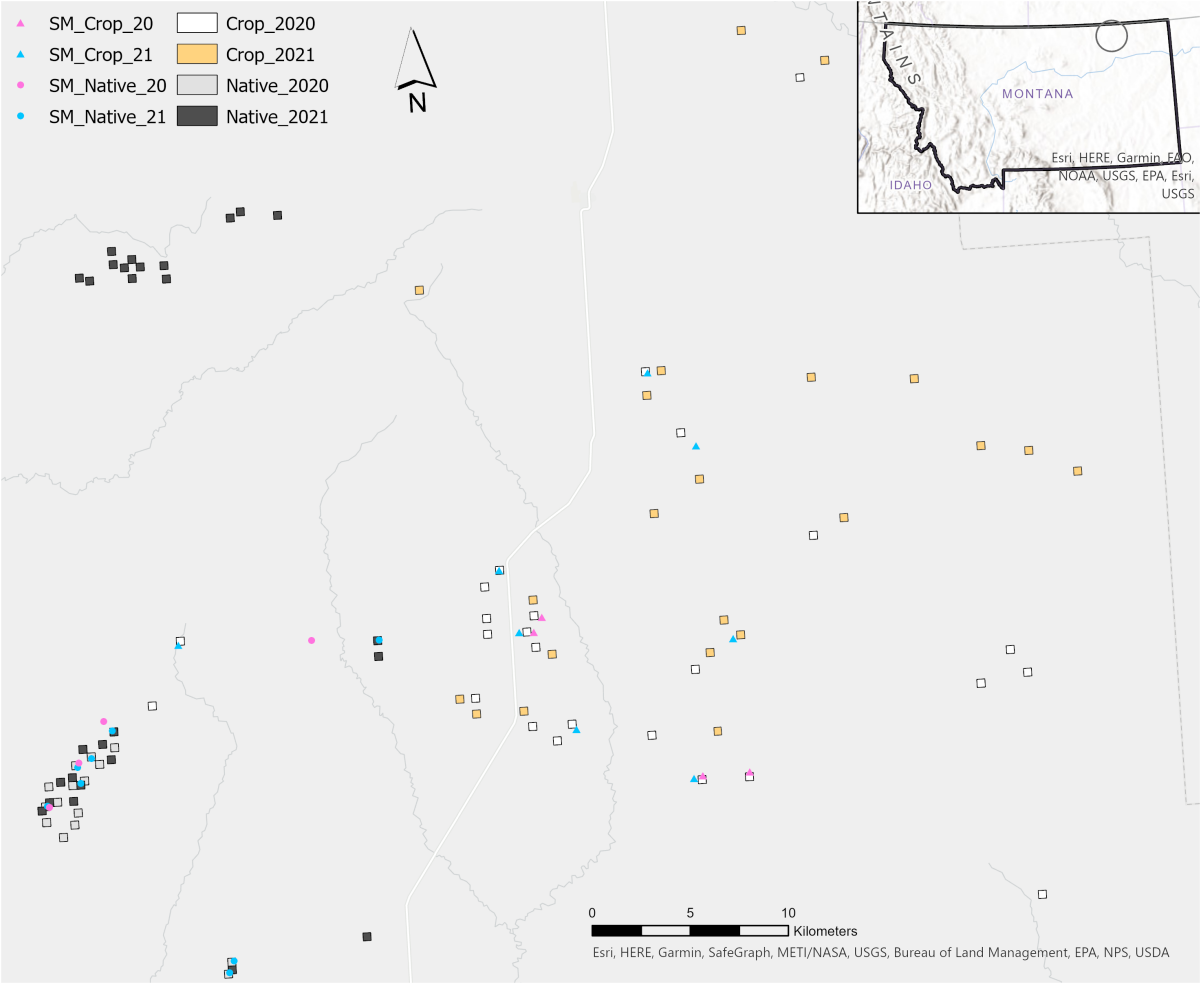
Map of study area illustrating song meter deployment sites (e.g., SM_Crop_20) and 16‐ha plots used for abundance surveys and nest searching on crop and native habitat sites in Valley County, Montana, 2020–2021. Clustering of native plots is due to patchy distribution of longspurs in native habitats as these plots were randomly selected from areas known to be occupied by thick‐billed longspurs.

Our study area incorporated native grassland in the western portion and cultivated fields in the eastern portion (Figure 2). Land ownership on native sites was a mix of federal lands and private ranches and included the Bitter Creek Wilderness Study Area. Native grassland in this region is classified as northern mixed‐grass prairie (Charboneau et al., 2013; Coupland, 1961). Cool‐season grasses dominated and common species included western wheatgrass (*Pascopyrum smithii*), needle‐and‐thread (*Hesperostipa comata*), prairie junegrass (*Koeleria macrantha*), green needlegrass (*Nassella viridula*), sandberg bluegrass (*Poa secunda*), and threadleaf sedge (*Carex filifolia*). One warm‐season grass, blue grama (*Bouteloua gracilis*), was present at some sites. Spikemoss (*Selaginella densa*) was locally abundant as well. Shrub cover (dominant species: silver sagebrush (*Artemisia cana*)) was low–moderate across the study area (Charboneau et al., 2013). This region contained large patches of arid soils, which provided habitat for longspurs and made them locally abundant in such areas. These specific sites consisted of aridic, well‐drained glacial soils of the Elloam series and had relatively low (<1000 kg ha^−1^) vegetation production potential (Lenard et al., 2006; Lipsey, 2015).

Crop types included primarily cereals (spring wheat) and pulse crops (pea and lentils, lentils often planted with flax). An additional crop type, summer fallow, was included in which spring wheat farming was conducted on a 50:50 rotation with 50% of the acreage fallowed each year to conserve soil moisture and nutrients (M. Sather, USFWS, pers. comm.). Less abundant crop types included oilseed (canola) and cover crops, and a mix of random seed types (i.e., spring wheat, flax, lentil, beets, and barley) planted during years a field was rested. Pulse crops are often planted between wheat rotations to add nitrogen, conserve soil moisture, and disrupt weeds, pests, and diseases (Long et al., 2014; Miller et al., 2002). Pulse crop rotations have replaced summer fallow over most of the region (M. Sather, USFWS, pers. comm.).

We used a geographic information system (ArcMap 10.7.1; ESRI, 2019) to select study plots in crop and native sites. We selected plots at two different levels: 64‐ha plots for initial surveys (Appendix S1), which were designed only to locate populations of breeding longspurs each year, and 16‐ha plots, which were generated within areas where longspurs occurred and were used to monitor longspur abundance and nest success (Figure 2).

Within initial plots (64 ha) was found to be occupied by longspurs (Appendix S1); we used ArcMap to generate smaller, 16‐ha survey plots within which we conducted abundance surveys and nest searching for the entire nesting season (Figure 2). Due to the patchy distribution of longspurs on native sites, our plot selection methods differed slightly for crop and native sites. We randomly selected between 20 and 30 occupied crop fields and generated a single 16‐ha plot within the center of each field. This ensured crop plots were ≥200 m from field edges, roads, and other plots. In occupied native sites, we delineated large patches of habitat by mapping the extent of occupied areas on foot with a GPS unit and later transferred this information to ArcMap. Patch edges were defined by the presence/absence of singing longspurs and were typically coupled with subtle changes in vegetation structure. We then overlaid a grid of 16‐ha cells over occupied patches and used ArcMap to randomly select between 20 and 30 cells from these patches. Only non‐adjacent cells were used to ensure plots were ≥200 m apart.

Our plot selection process in crop sites ensured that the distribution of crop types surveyed was representative of the study area, although it was limited to the land parcels where we were able to obtain permission to conduct sampling. All crop types except summer fallow were operationally similar throughout the breeding season. Crop sites, regardless of crop type, were structurally similar in April prior to seeding with all fields containing residual stubble (10–15 cm) from the previous year's crop. Timing of seeding was variable depending on land ownership but began in April and was completed by early June. Some fields were seeded directly into the previous year's stubble (no till), but most fields were disked prior to seeding (minimum till). Most producers conducted a weed control spray immediately prior to seeding. Post‐emergence herbicide applications were administered in most fields in June. In all summer fallow fields, tilling was typically conducted in unplanted portions two to three times between May and July for weed control. Some producers also used weed control spray in summer fallow fields. Harvest of all crops occurred in August or September, after the nesting season was complete. Because crop types were operationally similar, our goal was to obtain a representative sample across the study region instead of focusing on a particular crop type, although spring wheat was the dominant crop on the landscape and was disproportionally represented in our study plots (Table 1; Table S1). All native sites were grazed by cow/calf pairs after May 15; while cattle use of specific plots could not be quantified, grazing pressure was similar across all native sites.

**TABLE 1:** Breakdown of crop types for plots in which abundance surveys were conducted for thick‐billed longspur in Valley County, Montana 2020–2021.^a^

2020			2021		
Wheat	13	54%	Wheat	15	60%
Summer fallow	4	17%	Summer fallow	8	32%
Lentil/flax	3	13%	Lentil/flax	2	8%
Cover crop	2	8%			
Pea	1	4%			
Canola	1	4%			
Totals	24	100%		25	100%

*Note*: Shown are the number of survey plots in each crop type and percentage of the total for each type.

^a^ Plots were randomly selected and are thus representative of the distribution of crop types on the landscape. Other than summer fallow, all crop types were operationally similar and nesting birds experienced similar phenologies and disturbance patterns across crop types.

### 2.2 Field methods

#### 2.2.1 Longspur settlement

We deployed 24 autonomous acoustic recorders (Wildlife Acoustics model SM4, Maynard, MA; hereafter “song meters”) to assess settlement patterns of territorial male longspurs on the breeding grounds. Song meter locations were not part of the site selection process described above, as they were deployed before longspurs had arrived and surveys could begin. We consulted local biologists and used observations from the USFWS Breeding Bird Survey (BBS) and eBird (Sauer et al., 2020; Sullivan et al., 2020) to identify specific points annually used by longspurs. We deployed 8 and 16 song meters in 2020 and 2021, respectively, with half (4 in 2020, 8 in 2021) in crop sites and half in native sites (Figure 2). We selected sites that were no more than 25 km apart to minimize regional variation in weather patterns between site types. No crop fields were planted during deployment; fields were structurally similar and contained 10‐ to 15‐cm‐tall stubble from the previous year's crop. In 2020, song meters were placed in fields that contained the following crops the previous year: spring wheat (3) and flax (1); in 2021: spring wheat (5), lentil (1), and summer fallow (1). We deployed song meters from April 6 to 29, 2020, and April 7 to 30, 2021, to capture territory establishment. In Montana, male longspurs arrive about 2 weeks before females (early April), but do not typically settle onto territories until late April (With, 2021).

We affixed song meters to 1.8‐m t‐posts at a height of 1.2 m and covered each microphone with an extra layer of foam to reduce recorded wind noise. We programmed song meters to collect a 3‐min recording every half hour starting 15 min before sunrise and ending by 9:00 a.m. to coincide with morning breeding choruses of longspurs (With, 2021), resulting in six 3‐min recordings collected each morning. Upon removal from the field, a trained technician manually reviewed each 3‐min recording and documented longspur presence. We excluded any recordings in which ≥25% of the recording length was obscured by wind or other noise.

#### 2.2.2 Longspur abundance

##### Abundance surveys

We conducted six rounds of line transect surveys within each 16‐ha survey plot from May 10 to July 15, 2020–2021. Survey rounds were separated by ≥5 days. Observers walked a U‐shaped line transect within each plot, starting 100 m inward from a randomly selected plot corner (Figure S1; modified from Golding & Dreitz, 2017; Igl & Johnson, 1997). We recorded perpendicular distance and direction from the transect line for each longspur seen or heard. Estimated distances were recorded in bins: 0–25, 26–50, 51–75, and 76–100 m. We walked at a pace of 2–3 km h^−1^ and completed each transect within 30 min. Observers were trained to avoid double‐counting birds. Surveys began 15 min before sunrise and were completed by 9:00 a.m. We did not conduct surveys if wind speed was >25 km h^−1^ or if it was raining. We recorded survey covariates including observer, cloud cover, temperature, wind speed, date, GPS starting point, and transect start/end times.

#### 2.2.3 Nest phenology, survival, and reproductive output

##### 2.2.3.1 Nest searching

We searched for nests within the same 16‐ha plots to assess reproductive effort throughout the entire nesting season (With, 2021). Nest searching was conducted from May 9 to July 22, 2020, and May 5 to July 8, 2021; searching began at sunrise and ended at 11:00 a.m. on days without precipitation, and observers were randomly assigned a group of plots to search each morning. Observers alternated between crop and native sites during subsequent days and used behavioral observations to find nests (Martin & Geupel, 1993; Winter et al., 2003). We observed longspurs from a distance of ≥30 m and moved to a new plot after 60 min if no female longspurs were detected. In addition, we supplemented behavioral nest searching with standard rope‐dragging methods (Klett et al., 1986; Koford, 1999).

##### 2.2.3.2 Nest monitoring

Upon finding a nest, we recorded the geographical coordinates and marked the nest location with 15‐cm bamboo stakes placed 2 m north and east of the nest to aid in relocation. Nests were checked every 2–4 days until fledging or failure (Martin & Geupel, 1993; Ralph, 1993). We recorded adult behavior, number of eggs and young, number of brown‐headed cowbird (*Molothrus ater*) eggs or nestlings, date, time, observer, time spent at nest, and any relevant notes. We aged nestlings according to developmental cues described in Jongsomjit et al. (2007) so the nest could be checked on predicted date of fledging. We considered a nest failed if eggs were gone before expected hatch date, if nestlings disappeared before nearing expected fledge date, or if dead nestlings or depredated eggs were found in or near the nest. A nest was considered successful if ≥1 chick fledged. We deemed nests successful if nearby adults were observed feeding fledglings, ≥1 fledgling was observed, territorial adults were present with food or directed aggressive behaviors toward observers, or fecal material was present and nestlings had reached the appropriate age to fledge (Jones et al., 2010; Ralph, 1993).

#### 2.2.4 Habitat conditions

We collected vegetation measurements at two spatial scales, the nest site and 16‐ha survey plot. Measures were collected at each nest within 3 days of fledge or expected fledge for failed nests. In addition, we randomly selected 3 and 10 habitat sampling points within survey plots in crop sites and native prairie sites, respectively. Vegetation conditions in crop sites were fairly homogenous and required fewer sampling points (Swicegood, 2022). We measured vegetation conditions three times throughout the longspur breeding season, once each in May, June, and July. At each sampling point, we recorded visual obstruction reading (VOR) in each cardinal direction from a distance of 4 m and a height of 1 m (Robel et al., 1970). We measured overlapping percent cover of grass, forb, shrub, litter, and bare ground within a 20 × 50 cm sampling frame at the sampling point and 4 locations 0.5 m from the point in each cardinal direction (Daubenmire, 1959). Cover was recorded within six percentage classes (0%, 1%–5%, 6%–25%, 26%–50%, 51%–75%, 76%–95%, and 96%–100%). We listed all plant species, lichen, and spikemoss, in order of decreasing abundance, found within a 2‐m radius of the point center. We measured litter depth (mm) in the northwest corner of the Daubenmire frame and recorded the species, distance (m), and height (cm) of the nearest shrub within 25 m (Pulliam et al., 2021).

### 2.3 Analyses

#### 2.3.1 Longspur settlement

We used multi‐season occupancy models with our song meter data to evaluate whether longspur settlement patterns differed between crop and native sites (Mackenzie, 2006; MacKenzie et al., 2003). We combined data from both years due to small sample sizes, and each day represented a primary period (24) while each 3‐min recording represented a secondary period (6). We fitted models using the “colext” function in R package “unmarked” (Fiske & Chandler, 2011; Kéry & Chandler, 2016) and used information theory to evaluate support for competing models representing hypotheses about detection probability, initial occupancy, and settlement patterns (MacKenzie et al., 2003). We evaluated support for our a priori models in a phased approach. First, we evaluated how well a fully parameterized model fit the data and estimated a variance inflation factor (*ĉ*) using the *mb.gof.test* in the R package “AICcmodavg” from 500 bootstrapped simulations (Mazerolle, 2020). We found evidence of moderate overdispersion (*ĉ* = 1.9) and inflated estimated standard errors by √ *ĉ*. We based subsequent model evaluation and inference on the quasi‐Akaike's information criterion adjusted for finite samples (QAIC_c_; Burnham & Anderson, 2002).

We then developed models that evaluated the effects of survey conditions on detection probability. Variables hypothesized to influence detection included daily precipitation, minimum daily temperature, minutes past sunrise, and Julian day. We evaluated a quadratic effect of minutes past sunrise because bird detections were previously found to be highest mid‐morning (With, 2021). We did not include an effect of observer because recordings were annotated after removal from the field by a single primary observer. Because all detection covariates we measured are known to affect the detectability of songbirds, we used a backward selection approach based on QAIC_c_ to eliminate uninformative parameters (Arnold, 2010; Montgomery et al., 2021; Pagano & Arnold, 2009). Models with large relative weights (*w*_*i*_) and QAIC_c_ values ≤2 from the best‐fit model were considered equally parsimonious (Arnold, 2010; Burnham et al., 2011; Devries et al., 2008). After we identified a parsimonious sub‐model for detection, it was retained in subsequent evaluations of occupancy and settlement.

Because some birds had already arrived at the study area prior to song meter deployment, we evaluated whether initial occupancy differed by habitat type (crop vs. native) before evaluating the effects of habitat type and Julian day on settlement probability. Our candidate set included two models for initial occupancy and five models for settlement probability, which included an interaction term for habitat type and day. We hypothesized that settlement rates would change over the season differentially by habitat type. We also hypothesized that probability of site abandonment would be low because longspurs are unlikely to abandon a breeding site (With, 2021). Therefore, we did not include any models with covariates on settlement probability. Model selection was again based on QAIC_c_; when supported models differed by one parameter, we considered this parameter uninformative (Arnold, 2010; Burnham & Anderson, 2002). We used empirical Bayes methods to derive estimates of latent occupancy, or the true proportion of sites occupied, from the most parsimonious model for each primary period from predicted posterior distributions using the “ranef” function in R package “unmarked” (Fiske & Chandler, 2011). All analyses were performed using R Statistical Software (v 4.1.2; R Core Team, 2021).

#### 2.3.2 Longspur abundance

We used open‐population distance sampling models to estimate longspur abundance in crop and native sites and assess whether abundance changed differentially over the breeding season (Royle et al., 2004; Sollmann et al., 2015). We fitted models using the “distsampOpen” function in R package “unmarked” (Fiske & Chandler, 2011) and used information theory to evaluate support for competing models representing hypotheses about detection, initial abundance, and trends in abundance over the breeding season (Sollmann et al., 2015). Data were analyzed separately for the 2 years because differences in weather and drought conditions were likely to produce different population responses.

We evaluated support for a priori models in a phased approach. First, we used null models with the “trend” dynamics parameterization to estimate the best‐fitting detection function and mixture type based on our data. We then evaluated how well a fully parameterized model fit the data and estimated a variance inflation factor (*ĉ*) using the *Nmix.gof.test* in the R package “AICcmodavg” from 500 bootstrapped simulations. We used the hazard rate function and Poisson mixture type for all subsequent models, inflating estimated standard errors by √ *ĉ* and basing model evaluation and inference on the quasi‐Akaike's information criterion adjusted for finite samples (QAIC_c_; Burnham & Anderson, 2002). We found evidence of moderate overdispersion (*ĉ* = 1.9 for 2020 data and *ĉ* = 1.7 for 2021 data).

We then developed models to evaluate the effects of survey conditions on detection probability. Variables hypothesized to influence detection probability included observer, wind speed, temperature, and start time (minutes past sunrise). We evaluated a quadratic effect of start time because bird detections are usually highest 1–2 h after sunrise (With, 2021). Initial screening indicated that detection was variable across observers, so we separated observers into two groups for each year (“high” and “low” detection rates) based on relative coefficient estimates from a full model to reduce the number of parameters in candidate models while retaining observer effects on detection. We used the backward selection approach described previously to eliminate uninformative parameters and identify a parsimonious sub‐model for detection probability, which was retained in subsequent evaluations of abundance and seasonal trends.

Finally, we developed a set of four a priori models to test the effect of habitat type on both initial abundance and seasonal trend. Model selection was again based on QAIC_c_ (Burnham & Anderson, 2002). We used Bayesian methods to derive true abundance estimates from the most parsimonious model for each survey round from predicted posterior distributions using the “ranef” function in R package “unmarked” (Fiske & Chandler, 2011).

#### 2.3.3 Nest phenology, survival, and reproductive output

##### 2.3.3.1 Nest phenology

For each nest, we calculated initiation date as the day the last egg was laid, although actual initiation of incubation is variable for passerines (Badyaev et al., 2003; Hébert, 2002). Initiation date was estimated based on clutch size, hatch date, or chick age and assuming an incubation period of 12 days (With, 2021). For nests found after clutch completion but destroyed before hatch, we assumed initiation to be 6 days prior to the midpoint of the active period. We plotted nest initiation dates to visualize patterns of nest initiation between crop and native sites and to assess differences between years.

##### 2.3.3.2 Nest survival

We used the nest survival model in program MARK to model daily nest survival rate (DSR), and we fitted models in the R package “Rmark” (Laake, 2013; Rotella et al., 2004; White & Burnham, 1999). We built and evaluated a set of 15 competing models representing a priori hypothesized relationships between DSR and habitat type (crop or native), nest initiation date, and year (2020, 2021). Our models included all combinations of habitat type, initiation date, and year. We also included a model with a quadratic effect of initiation date because other studies have shown DSR to be higher or lower mid‐season (Skagen et al., 2018; Weintraub et al., 2016). We predicted that DSR may exhibit a pseudo‐threshold response in crop sites only, being low for nests initiated early and leveling off after fields were planted. Therefore, we included a model with a pseudo‐threshold effect of initiation date and one including an interaction term with habitat type. We evaluated the relative support of models using Akaike's information criterion corrected for finite sample size (AIC_c_; Burnham & Anderson, 2002). To estimate nest survival probability, we used a 26‐day nesting cycle beginning with the start of the laying period and multiplied DSR for each daily interval over a 25‐day period from nest initiation to fledging (e.g., DSR^25^ for constant model). We calculated standard error for nest survival estimates using the Delta method (Powell, 2007).

##### 2.3.3.3 Reproductive output

We calculated an index of nest density for each plot by dividing the number of nests located in each plot by the total search effort (hours) for that plot. We report the mean and standard deviation of relative nest density for each habitat type. Incidental nests located outside of survey plots and nests found via rope‐dragging methods were excluded from this calculation. We were unable to account for detectability of nests with behavioral search methods, and it is possible that detectability differed in crop and native sites. Detectability almost certainly differed by observer (Diefenbach et al., 2003; Giovanni et al., 2011); therefore, observers were rotated through different plots each day.

We tabulated maximum clutch size for all nests with known fates and the number of young fledged per successful nest. The number of young fledged was recorded as the number of chicks present 8–10 days after hatching, the typical fledging time for longspurs (With, 2021). We developed a set of generalized linear models to analyze the effects of habitat type and initiation date on the number of young fledged per successful nest using a Poisson distribution with a log link. Our five candidate models included an interaction term to test if the number of young fledged differed by both habitat type and initiation date. Nests were removed from analysis if the number of young fledged was unknown. We evaluated relative model support using AIC_c_ (Burnham & Anderson, 2002) and used the best‐fitting model to estimate the number of young fledged per successful nest.

#### 2.3.4 Habitat conditions

We used generalized linear models to test hypotheses that specific vegetation attributes differed significantly between crop and native sites, longspur habitat changed structurally over the summer as plants grew, and such changes were more extreme in crop sites than in native sites. Variables included VOR, bare ground cover, grass and forb cover, litter cover, and litter depth. For proportional response data (e.g., percent coverages), we used the binomial distribution and logit link function to fit GLMs (Chen et al., 2017). For all other vegetation measures, including VOR and litter depth, we used the identity link and log‐transformed the response variables to meet the assumptions of linear regression (Dunn & Smyth, 2018). For each habitat variable, we built and evaluated the same set of five competing models representing a priori hypothesized relationships between habitat type and survey round. We evaluated relative model support using AIC_c_. We based inferences on effect sizes from a single top model and calculated model‐averaged estimates when models shared support (ΔAIC_c_ ≤ 2; Burnham & Anderson, 2002).

## 3 RESULTS

### 3.1 Longspur settlement

We deployed song meters at 8 and 16 sites in 2020 and 2021, respectively, half in crop fields and half in native sites. Due to equipment malfunction and failure of longspurs to establish territories at some sites, we were able to obtain data from two song meters in native sites and four song meters in crop sites in 2020, and seven song meters in native sites and seven song meters in crop sites in 2021. Overall, we collected >37 h of useable recordings in 2020 and >100 h in 2021.

#### 3.1.1 Detection probability

The top model for detection probability contained an effect of Julian day, minimum temperature, and a quadratic effect of minutes past sunrise (QAIC_c_ *w*_*i*_ = 0.97; Table 2). Detection probability increased with Julian day (*β* = 0.99 ± 0.13 SE) and increased in response to minimum temperature (*β* = 0.08 ± 0.02). Detection probability was highest at ~90–100 min past sunrise, or 1.5 h after sunrise (Figure S2).

**TABLE 3:** Model selection results from open‐population distance models for detection probability (*p*), initial abundance (*N*_1_), and seasonal trends (*λ*) of thick‐billed longspur populations.

Model	*K*	QAIC_c_	ΔQAIC_c_	QAIC_c_Wt
Detection 2020^b^				
*p*(Obs)	6	1010.17	0.00	0.62
*p*(Obs + Temp)	7	1011.32	1.15	0.35
*p*(Obs + Temp + Start^2^)	9	1016.30	6.13	0.03
*p*(Obs + Wind + Temp + Start^2^)	10	1019.05	8.87	0.01
*p*(.)	5	1026.12	15.95	0.00
Initial abundance, Trend 2020^c^				
*N*_1_ (Habitat) *λ* (Habitat)	8	994.99	0.00	0.91
*N*_1_ (Habitat) *λ* (.)	7	999.56	4.56	0.09
*N*_1_ (.) *λ* (Habitat)	7	1010.00	15.01	0.00
*N*_1_ (.) *λ* (.)	6	1010.17	15.18	0.00
Detection 2021				
*p*(Obs)	6	1294.38	0.00	0.53
*p*(Obs + Start)	7	1295.48	1.10	0.31
*p*(Obs + Temp + Start)	8	1297.93	3.55	0.09
*p*(.)	5	1299.28	4.90	0.05
*p*(Obs + Temp + Start^2^)	9	1300.67	6.29	0.02
*p*(Obs + Wind + Temp + Start^2^)	10	1303.81	9.43	0.00
Initial abundance, Trend 2021				
*N*_1_ (.) *λ* (.)	6	1294.38	0.00	0.54
*N*_1_ (.) *λ* (Habitat)	7	1295.94	1.56	0.25
*N*_1_ (Habitat) *λ* (.)	7	1297.08	2.70	0.14
*N*_1_ (Habitat) *λ* (Habitat)	8	1298.68	4.30	0.06

*Note*: Data come from line transect surveys conducted in Valley County, Montana, from May to July, 2020–2021.^a^ The number of parameters (*K*), QAIC_c_ values, ΔQAIC_c_ values, and model weights (QAIC_c_Wt) are reported.

^a^ We surveyed 46 plots in 2020 (24 in crop, 22 in native) and 50 plots in 2021 (25 in crop, 25 in native) six times from May to July.

^b^ Detection covariates include an effect of observer (Obs), temperature at survey start time (Temp), a quadratic effect of minutes past sunrise for survey start time (Start), and wind (Wind).

^c^ We tested for an effect of crop or native habitat type (Habitat) on both initial abundance and seasonal trend.

#### 3.1.2 Initial occupancy and settlement probability

**FIGURE 3:**
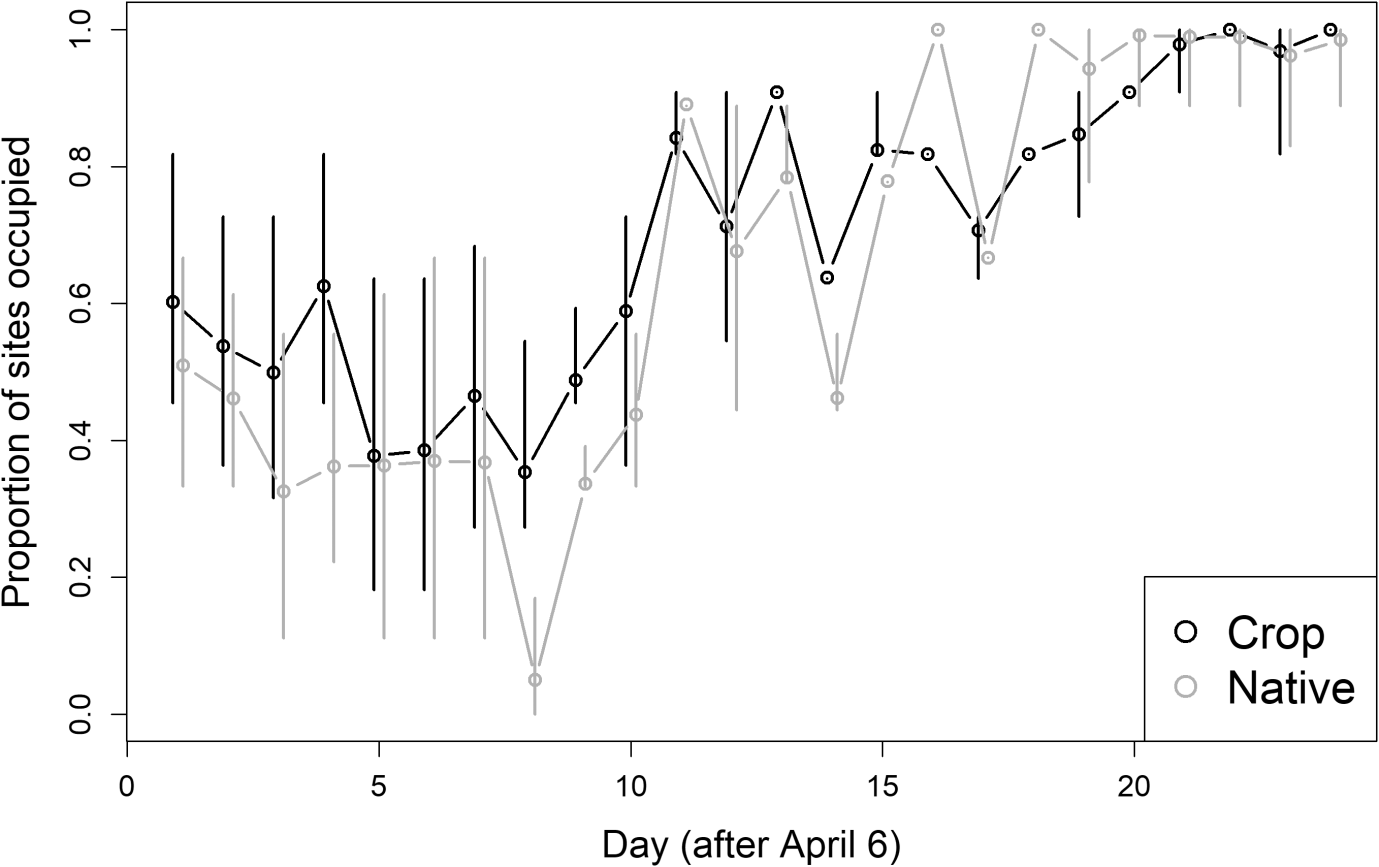
Estimated occupancy during each day (April ~7 to April 30) of longspur settlement in Valley County, Montana, 2020–2021. Results are from dynamic occupancy models and whiskers indicate 95% confidence intervals.

We found no evidence for an effect of habitat type on initial occupancy with the null model carrying virtually all support (QAIC_c_ *w*_*i*_ = 0.98; Table 2). We found no evidence that settlement probability differed by habitat type with the model containing an effect of Julian day carrying virtually all support (QAIC_c_ *w*_*i*_ = 0.98; Table 2). Settlement probability increased for both habitat types with Julian day (*β* = 2.24 ± 0.68). Derived estimates of latent occupancy for both crop and native sites increased from 0.52 (±0.17 SE) on April 7 to 0.99 (±0.01) on April 30 (Figure 3).

### 3.2 Longspur abundance

In 2020, we conducted 287 longspur abundance surveys at 24 crop sites and 22 native sites from May 14 to July 19. We observed 5.4 ± 4.4 (mean ± SD) male longspurs in crop sites and 4.2 ± 3.3 in native sites. In 2021, we conducted 325 surveys at 25 crop sites and 25 native sites from May 10 to July 14. We observed an average of 3.8 ± 3.2 and 3.2 ± 2.3 males per plot in crop and native sites, respectively.

#### 3.2.1 Detection probability

For both years, the top model contained an effect of observer group (Table 3). Detection probability was lower for observer group 2 (*β* = −1.67 ± 0.54 SE in 2020; *β* = −0.95 ± 0.39 in 2021; Figure S3). Confidence intervals for the effect sizes for other covariates on detection overlapped 0; therefore, only observer group was retained in subsequent abundance modeling (Arnold, 2010).

**TABLE 2:** Model selection results from multi‐season occupancy models for detection probability (*p*), initial occupancy (*ψ*_1_), and settlement probability (𝛾) from acoustic data collected over a 24‐day period in Valley County, Montana, during the month of April, 2020 and 2021.^a^

Model	*K*	QAIC_c_	ΔQAIC_c_	QAIC_c_Wt
Detection^b^				
*p*(Time^2^ + Mintemp + Day)	9	834.02	0.00	0.97
*p*(Time^2^ + Mintemp + Day + Precip)	10	841.04	7.02	0.03
*p*(.)	5	990.06	156.04	0.00
Initial occupancy^c^				
*ψ*_1_ (.)	9	834.02	0.00	0.98
*ψ*_1_ (Habitat)	10	842.15	8.13	0.02
Settlement				
𝛾 (Day)	10	824.74	0.00	0.98
𝛾 (.)	9	834.02	9.29	0.01
𝛾 (Day + Habitat)	11	834.36	9.63	0.01
𝛾 (Habitat)	10	842.40	17.67	0.00
𝛾 (Day × Habitat)	12	846.58	21.84	0.00

*Note*: The number of parameters (*K*), QAIC_c_ values, ΔQAIC_c_ values, and model weights (QAIC_c_Wt) are reported.

^a^ 20 total recorders were deployed over the two study years, 9 in crop sites and 11 in native sites. Due to small sample sizes, data were combined over both years.

^b^ Detection covariates include a quadratic effect of minutes past sunrise (Time), daily minimum temperature (Mintemp), daily precipitation (Precip), and day of the survey period (Day).

^c^ Covariates for initial occupancy include crop or native habitat type (Habitat); covariates for settlement probability include crop or native habitat type (Habitat) and day of survey period (Day).

#### 3.2.2 Initial abundance and seasonal trends

**FIGURE 4:**
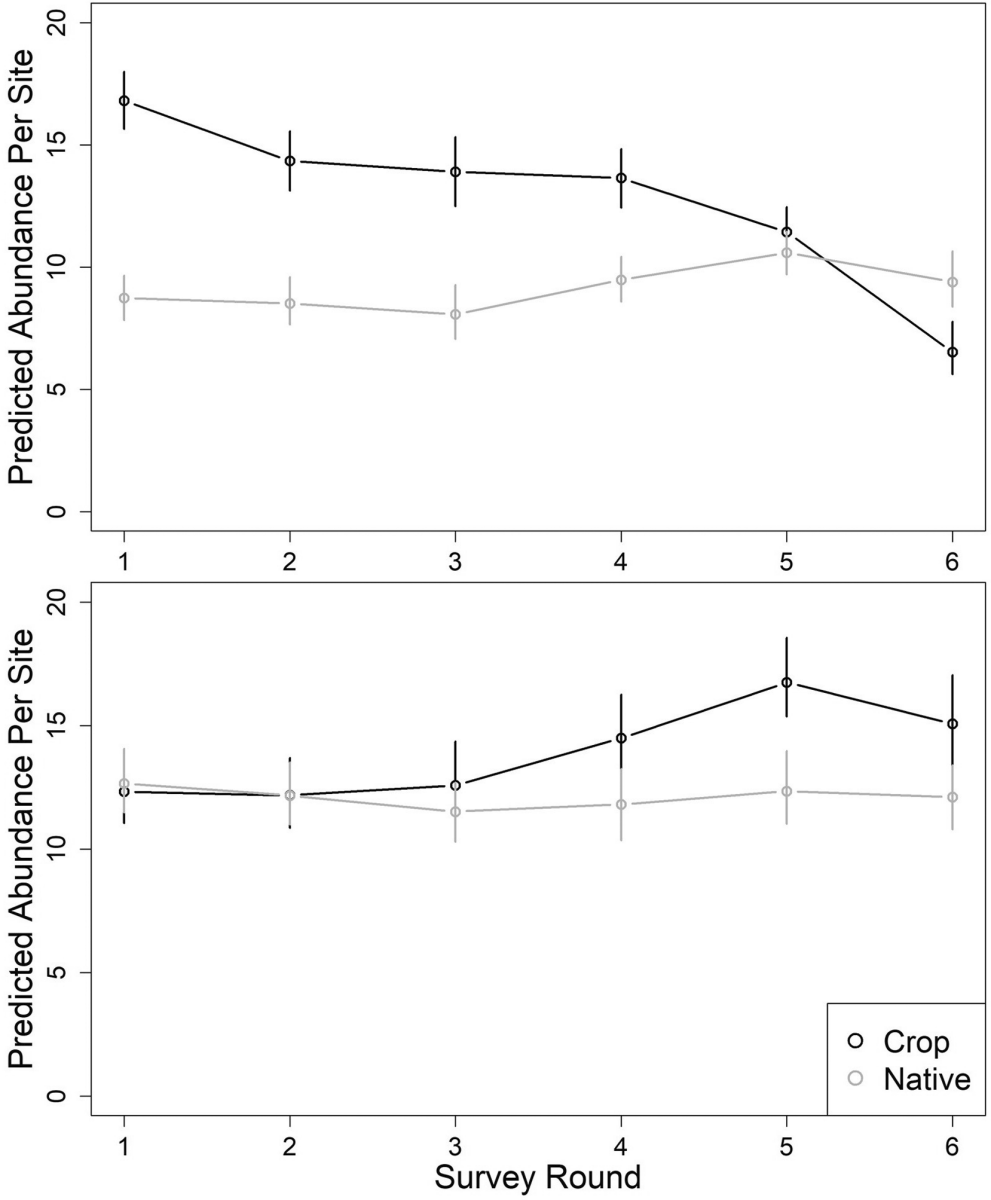
Estimated plot‐level abundance of thick‐billed longspurs in both crop and native sites in Valley County, Montana, 2020 (top) and 2021 (bottom). Whiskers depict 95% confidence intervals. Survey rounds were evenly spaced between May 10 and July 15 each year.

We found support for an effect of habitat type on both initial abundance and seasonal trend for data collected in 2020 (QAIC_c_ *w*_*i*_ = 0.91; Table 3). Expected initial abundance in crop sites was 17.4 ± 4.1 SE birds per plot and the estimated seasonal trend was *λ* = 0.84 ± 0.04, indicating that abundance decreased by 16% over the season. Expected initial abundance in native sites was 8.6 ± 2.0 birds per plot and increased slightly during the season (*λ* = 1.02 ± 0.05). Derived estimates of true abundance for crop sites decreased from 16.8 (95% CI = 15.7–18.0) during the first survey round to 6.5 (5.6–7.8) during the sixth round. Derived abundance estimates for native sites were 8.7 (95% CI = 7.8–9.7) during the first survey round and 9.4 (8.4–10.7) during the sixth round (Figure 4).

In 2021, we found no support for an effect of habitat type on either initial abundance or seasonal trend, with the null model carrying the most support (QAIC_c_ *w*_*i*_ = 0.54; Table 3). Because of model uncertainty, we averaged results across all four supported candidate models. Expected initial abundance was similar in crop and native sites (12.5 ± 3.3 SE) and seasonal population sizes did not change much during the season (*λ* = 1.03 ± 0.04 SE in crop sites; 1.01 ± 0.04 in native sites). Derived estimates of true abundance for crop sites increased slightly from 12.3 (95% CI = 11.1–13.3) during the first survey round to 15.1 (13.2–17.0) during the sixth round. Derived estimates of true abundance for native sites were fairly stable across the season: 12.7 (95% CI = 11.5–14.1) during the first survey round and 12.1 (10.8–13.4) during the sixth round (Figure 4).

### 3.3 Nest phenology, survival, and reproductive output

In 2020, we conducted nest searching in 47 (25 crop and 22 native) plots. We found nests in 88% of crop plots and 82% of native plots. In 2021, we conducted nest searching in 55 (27 crop and 28 native) plots. We found nests in 63% of crop plots and 68% of native plots (Table S1). We located 240 total longspur nests, 111 in crop sites and 129 in native sites. Of these, 174 were located using behavioral cues of adults, 14 using rope‐dragging methods, and 52 were incidental finds while observers were conducting other fieldwork. We spent 515 h behavioral searching in crop sites and 421 h behavioral searching on native sites, for a total of 936 h nest searching using behavioral cues. Using two to three observers, we spent 76.5 person‐hours rope dragging in crop fields and 22.5 person‐hours rope dragging in native sites, for a total of 99 rope‐dragging person‐hours.

Of the 240 nests, 222 had known fates (96 crop and 126 native plots; Table S2). For the 18 remaining nests, we were unable to determine nest fate due to either conflicting clues at the nest site or weather events/farming operations preventing timely nest checks near expected fledge date. We were able to estimate the number of young fledged for 87 successful nests, 41 crop and 46 native (Table S2). Apparent nest success was 44% (*n* = 96) in crop sites and 37% (*n* = 126) in native sites. Predation was the main cause of nest failure in both crop and native sites (Table 4). Other causes included weather, farming operations (crop only), and abandonment. Brown‐headed cowbird (*Molothrus ater*) parasitism rates were 2.1% and 8.7% of crop (*n* = 96) and native (*n* = 126) nests, respectively.

**TABLE 4:** Apparent causes of nest failure for thick‐billed longspurs in Valley County, Montana, 2020–2021.

Cause of nest failure	2020		2021	
	Crop (%)	Native	Crop (%)	Native (%)
Predation	54	70%	69	79%
Abandonment^a^	11	21%	12.5	21%
Weather^b^	18	9%	6	0%
Farming operations	18	N/A	12.5	N/A

*Note*: Percentages are based on 40 failed crop nests and 46 failed native nests in 2020 and 14 failed crop nests and 34 failed native nests in 2021. Determination was based on sign around the nest near time of failure; failed nests with uncertainty regarding cause were removed from these calculations.

^a^ Abandonment often occurred after weather or partial predation events in both site types.

^b^ Weather events included flooding, hail, or storm damage which resulted in nest destruction or destruction of nest contents.

#### 3.3.1 Nest phenology

**FIGURE 5:**
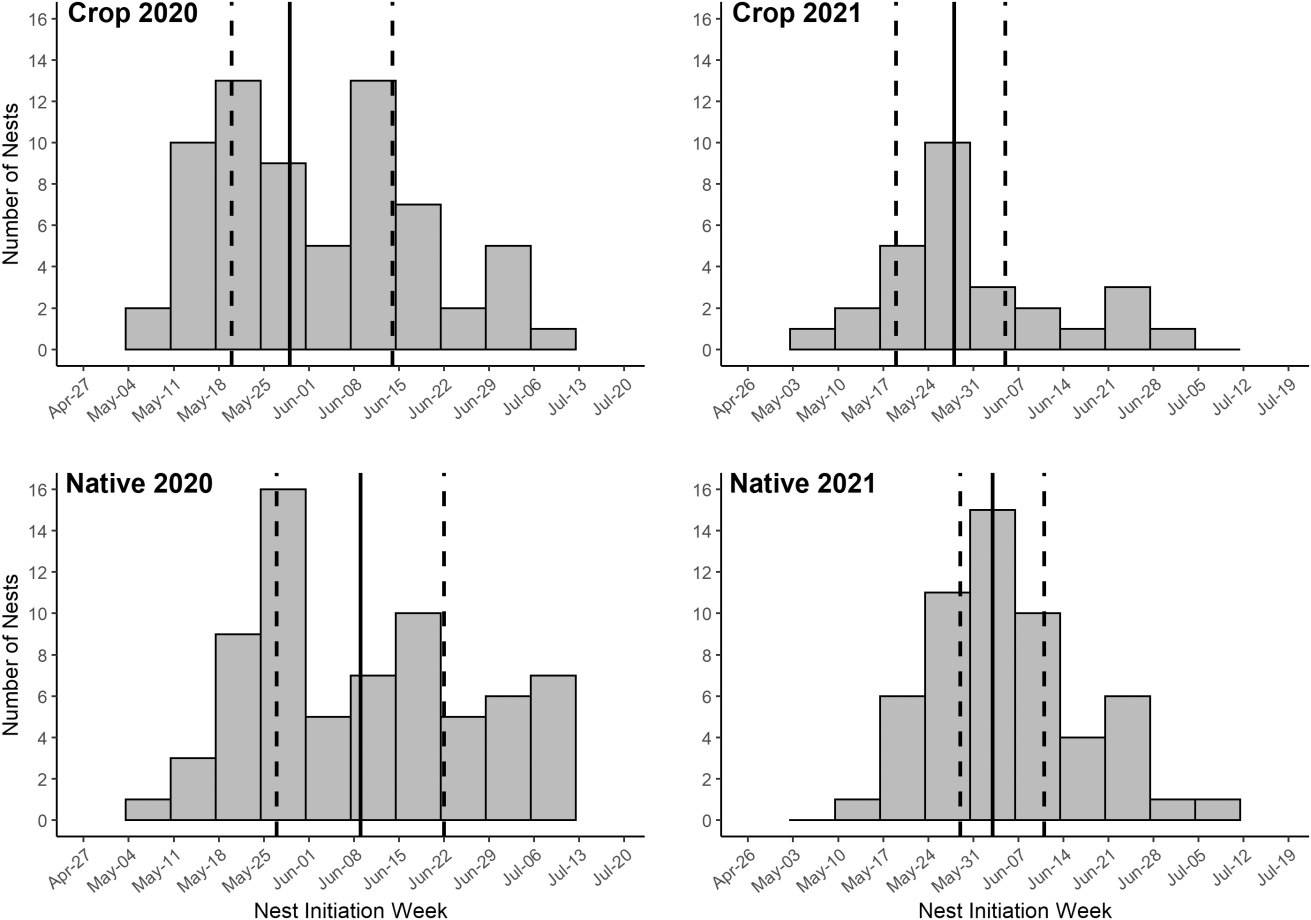
Estimated nest initiation dates in both crop and native sites for 222 thick‐billed longspur nests found in Valley County, Montana, 2020–2021. Results are based on 139 nests in 2020 (68 crop, 71 native) and 83 nests in 2021 (28 crop, 55 native). Overall nest initiation patterns were similar between crop and native sites given the year; 2020 was relatively cool and wet and 2021 was a drought year. Solid line represents the median nest initiation date and dotted lines represent the first and third quartiles.

Patterns of nest initiation were similar within crop and native sites each year, but median initiation dates in native sites were 6–11 days later than median dates in crop sites (Figure 5). In addition, the first and third quartiles were 6–10 days later in native sites. In 2020, median initiation date was May 29 (IQR = 25 days, *n* = 68) and June 9 (IQR = 26 days, *n* = 71) in crop and native sites, respectively. Longspurs nested through mid‐July and there were two prominent peaks in nest initiation. In 2021, median date of initiation was May 28 (IQR = 17 days, *n* = 28) in crop sites and June 3 (IQR = 13 days, *n* = 55) in native sites. Nesting slowed significantly in early July, and there was only one main peak in nest initiation. Notably, the interquartile distance for initiation dates was 32% shorter in crop sites and 50% shorter in native sites during the 2021 drought.

#### 3.3.2 Nest survival

The null model of constant daily nest survival was the best supported in the candidate set (AIC_c_ *w*_*i*_ = 0.18; Table 5). Models including effects of habitat type, year, and initiation date, including models with different functional forms of initiation date, had approximately equal support as the null model, indicating that these parameters were uninformative. Average daily nest survival estimated from the null model was 0.944 ± 0.005 SE and estimated nest survival over the 26‐day exposure period (DSR^25^) was 0.236 ± 0.028.

**TABLE 5:** Model selection results from nest survival models predicting effects on daily nest survival rate (DSR) of 222 thick‐billed longspur nests in Valley County, Montana, 2020–2021.

Model	*K*	AIC_c_	ΔAIC_c_	AIC_c_Wt
S(.)	1	746.38	0.00	0.18
S(Initiation)	2	747.13	0.75	0.12
S(Initiation^2^)	2	747.43	1.06	0.11
S(Year)	2	747.46	1.09	0.10
S(ln(Initiation))	2	747.99	1.62	0.08
S(Habitat)	2	748.35	1.97	0.07
S(Year + Initiation)	3	748.38	2.00	0.07
S(Year × Initiation)	4	748.86	2.48	0.05
S(Habitat + Initiation)	3	748.98	2.60	0.05
S(Habitat × Initiation)	4	749.04	2.67	0.05
S(Habitat × ln(Initiation))	4	749.39	3.01	0.04
S(Habitat + Year)	3	749.39	3.01	0.04
S(Habitat + Year + Initiation)	4	750.16	3.79	0.03
S(Habitat × Year)	4	750.90	4.52	0.02
S(Habitat × Year × Initiation)	8	752.98	6.60	0.01

*Note*: Included are the effects of nest initiation date, nest initiation date^2^, year, and habitat type (crop, native). The number of parameters (*K*), AIC_c_ values, ΔAIC_c_ values, and model weights (AIC_c_Wt) are reported.

#### 3.3.3 Reproductive output

Relative nest density (±SD) was 0.153 ± 0.215 nests/hour/plot in crop sites and 0.233 ± 0.317 nests/hour/plot in native sites. Mean clutch sizes ±SD were 3.5 ± 0.8 and 3.3 ± 0.8 for nests occurring in crop and native sites, respectively. The mean number of young fledged per successful nest was 3.0 ± 1.1 SD in crop and 2.8 ± 0.9 in native sites (Table S2). The null model was the best‐supported model in our candidate set of generalized linear models for number of young fledged per successful nest (AIC_c_ *w*_*i*_ = 0.49; Table 6), indicating that neither nest initiation date nor habitat type was related to the number of young fledged. Models including the effects of habitat type and initiation date had approximately equal support as the null model, indicating that these parameters were uninformative. Estimated from the null model, the average number of young fledged per successful nest in both crop and native sites was 2.90 ± 0.18 SE.

**TABLE 6:** Model selection results from generalized linear models predicting number of chicks fledged from 87 successful thick‐billed longspur nests in Valley County, Montana, 2020–2021.

Model	*K*	AIC_c_	ΔAIC_c_	AIC_c_Wt
Null	1	286.32	0.00	0.49
Habitat	2	287.98	1.66	0.21
Initiation date	2	288.18	1.86	0.19
Habitat + Initiation date	3	289.84	3.52	0.08
Habitat × Initiation date	4	292.01	5.68	0.03

*Note*: We used a Poisson distribution and a log link and included the effects of nest initiation date and habitat type (crop, native). The number of parameters (*K*), AIC_c_ values, ΔAIC_c_ values, and model weights (AIC_c_Wt) are reported.

### 3.4 Habitat conditions

We observed significant differences in vegetation conditions between crop and native sites that varied across survey rounds (Appendix S2). Visual obstruction reading (VOR) estimates (cm ± SE) in 2020 changed from 0.81 ± 1.42 in May to 17.81 ± 1.43 in July in crop sites and from 1.95 ± 1.51 to 2.61 ± 1.46 in native sites. In 2021, VOR estimates (cm ± SE) changed from 0.72 ± 1.35 to 1.48 ± 1.35 in crop sites and from 0.68 ± 1.34 to 0.28 ± 1.34 in native sites. Bare ground coverage was significantly lower on native sites than on crop sites during both years. Estimated bare ground (% ± SE) in 2020 was 45 ± 6 in crop and 10 ± 4 in native sites. In 2021, estimated bare ground was 42 ± 6 in crop and 14 ± 4 in native sites.

Estimated litter coverage in 2020 (% ± SE) was 25 ± 5 in crop sites and 8 ± 3 in native sites. In 2021, estimated litter coverage was 26 ± 5 in crop and 11 ± 3 in native sites. Estimated litter depth in 2020 (mm ± SE) changed from 4.66 ± 1.30 in May to 0.58 ± 1.31 in July in crop sites and from 1.57 ± 1.34 to 1.05 ± 1.32 in native sites. In 2021, estimates changed from 2.75 ± 1.12 in May to 1.35 ± 1.12 in July in crop sites and from 1.01 ± 1.12 to 0.91 ± 1.12 in native sites. Models of residual, forb, and grass cover indicated that these vegetation conditions were similar across habitat types and survey rounds (Appendix S2).

## 4 DISCUSSION

### 4.1 Crop fields as potential traps

Collectively, our results did not support the hypothesis that cereal and pulse crop fields are ecological traps for breeding thick‐billed longspurs because, compared with longspur use of native grassland sites, there was no evidence of preference for crop habitat or of suppressed reproduction in crop sites. Specifically, settlement patterns of singing males were similar between crop and native sites, indicating that selection cues and preference of longspurs were also similar between habitat types. Also, our nest density index was 29% lower in crop sites, providing no evidence for preferential nesting in crop habitats. Furthermore, nest survival, average clutch size, and the number of young fledged were similar between crop and native sites, providing no evidence for suppressed reproduction in crop fields. Our results indicate that crop fields provide additional nesting opportunities for a species with a naturally restricted native range.

Although some nests were destroyed by farm machinery in crop sites, longspurs are quick to renest (<10 days; Felske, 1971; Mickey, 1943; With, 2021), and we often found new nests close to failed nest locations. While the most common cause of nest failure in both habitat types was predation, higher predation rates on native sites resulted in similar overall nest survival rates to crop sites. Known predators of longspur nests in native sites include Richardson's ground squirrels (*Urocitellus richardsonii*), voles (*Microtus* spp.), badgers (*Taxidea taxus*), striped skunks (*Mephitis mephitis*), red fox (*Vulpes vulpes*), swift fox (*Vulpes velox*), coyotes (*Canis latrans*), long‐tailed weasel (*Mustela frenata*), mice (*Peromyscus* spp.), bullsnakes (*Pituophis catenifer*), garter snakes (*Thamnophis* spp.), hawks (*Buteo* spp.), and American crows (*Corvus brachyrhynchos*; Felske, 1971; Mickey, 1943; Sedgwick, 2004; With, 1994). Common predators of passerine nests in crop fields include skunks, raccoons (*Procyon lotor*), red foxes, snakes, and ground squirrels (Basore et al., 1986; Ribic et al., 2012).

In contrast to expectations, some farming activities, including rolling (field leveling) and spraying, did not result in nest damage or abandonment. Longspur nest bowls were constructed below the soil surface, and we found that farming activities that did not disturb the soil did not negatively affect nests regardless of nest stage (*n* = 9 nests in leveled fields). Although nests were active during herbicide application, spraying did not directly result in losses of eggs or nestlings. However, we did not assess potential indirect effects of herbicide or fertilizer spraying on nestling growth rates or subsequent fledgling survival.

Flooding and hail destroyed nests in both crop (*n* = 8) and native sites (*n* = 4). Nest abandonment was often due to partial predation, weather, brown‐headed cowbird parasitism, and possibly frequent disturbance by predators or perceived predators. On a few occasions in native sites (*n* = 3 nests), we found all nestlings apparently uninjured but laying outside the nest. These nestlings never survived and were never returned to the nest by adult longspurs. We suspect this to be the activity of brown‐headed cowbirds or other passerine nest predators (Pietz et al., 2012; Pietz & Granfors, 2000).

### 4.2 Longspur abundance and use of crop fields

Our prediction that suitability of crop fields as nesting habitats would decline over the growing season was supported by observed trends in longspur abundance. We are not aware of other studies that specifically investigated how attractiveness of cereal or pulse crop fields changed over the breeding season for thick‐billed longspur, but similar trends have been observed for Eurasian skylarks (*Alauda arvensis*) nesting in cereal crops (Donald & Vickery, 2000) and for horned larks (*Eremophila alpestris*) nesting in winter wheat fields (Davis et al., 2020), both of which prefer short‐stature vegetation for nesting. Longspur abundance declined in crop sites during 2020 as vegetation height increased, but we did not observe the same trend in 2021 when a drought suppressed crop growth. Because rates of nest abandonment were ubiquitously low, declining abundances of longspurs imply reduced nesting attempts in crop sites in 2020, although we could not confirm this with unmarked birds. Therefore, precipitation and associated vegetation growth appeared to mediate longspur abundance and use of cereal and pulse crop fields. Curtailment of the nesting season in these fields as crop height increases may reduce season‐long fecundity for birds nesting in crop fields. Furthermore, annual variation in timing of seeding coupled with drought effects on vegetation may increase the unpredictability of crop habitat among years.

While we found similar numbers of nests in crop and native sites in 2020, we found approximately half the number of nests in crop sites compared to native sites in 2021. It is possible that during drought years, fewer longspurs attempt to nest in crop fields. Given that longspur abundances were similar between crop and native sites in 2021, yet we found half the number of nests, we speculate that drought may have reduced suitability for nesting in crop sites that year. This would support our expectation that crop fields may be less predictable than native grassland as nesting habitats. While not an ecological trap, high variability of crop fields as nesting habitats between years may lead to population declines if a large proportion of the longspur population establishes breeding territories in crop sites. Although an interesting avenue for further research, we were unable to distinguish true nest density from our ability to detect nests.

We found relatively fewer nests in summer fallow fields compared to spring wheat and lentil fields (*n* = 11 total nests in summer fallow fields). The fallow portions of summer fallow fields pose additional hazards to ground‐nesting birds because they are tilled multiple times during the breeding season. Therefore, nests in unplanted portions of summer fallow fields have a higher risk of being destroyed later in the season, whereas in other crop types, longspurs were only vulnerable to this particular hazard during the early nesting season. It is also possible that frequent tilling of fallow fields results in different soil conditions, generating different invertebrate resource availability than that found in minimum‐tilled fields (Kladivko, 2001; Stinner & House, 1990).

### 4.3 Timing of nesting

We found that median nest initiation occurred 6–11 days earlier in crop sites despite similar settlement patterns for the two habitat types. Longspurs appeared to shift timing of nesting in crop sites, and perhaps this phenological shift is beneficial in habitat that changes to become unsuitable late in the breeding season. In native prairie habitats, longspurs select territories on south‐facing slopes during the early breeding season where snow melts and the ground warms faster (Felske, 1971; Greer, 1988). Bare ground cover was higher in crop sites than native sites throughout the breeding season, and exposed soils warm faster than vegetated soils (Shaffer et al., 2019; Song et al., 2013). Earlier warming of crop fields may allow earlier nest initiation and egg laying resulting from favorable microclimatic conditions or an earlier invertebrate food supply (Felske, 1971; Greer & Anderson, 1989). However, we did not assess thermal or other microclimatic conditions at nests. Furthermore, the range of nest initiation dates and therefore length of nesting period was significantly shorter during the drought of 2021. Longspurs are known to forego nesting or experience lower reproductive success during periods of extreme drought (Felske, 1971). Our results indicate that longspurs may initiate nests earlier in crop than native sites but experience a shorter breeding period in both site types during drought.

### 4.4 Data limitations

A broader demographic analysis including seasonal adult, annual adult, juvenile, and post‐fledging survival rates would increase our ability to identify population sources and sinks and more fully test the ecological trap hypothesis. Our estimates for nest survival and the number of young fledged per successful nest are similar to estimates reported in other studies of thick‐billed longspur and similar species in native prairie habitats, including horned lark and chestnut‐collared longspur (*Calcarius ornatus*; Gaudet et al., 2020; Lloyd & Martin, 2005; Mahoney & Chalfoun, 2016; Pulliam et al., 2021; Reintsma et al., 2022; Sedgwick, 2004; Shaffer et al., 2019). Because double brooding in longspurs can be affected by seasonal habitat conditions that vary differentially in crop fields and native prairie, future research should evaluate the relative contribution of each habitat type to overall fecundity (i.e., fledglings per female per season) and how this varies between years. To better understand mechanisms driving longspur population declines, additional information is needed on vital rates across life stages (e.g., renesting rates, juvenile survival, and adult survival). Grassland birds are highly sensitive to variation in annual adult survival (Perlut et al., 2008; Sedgwick, 2004), however, low site fidelity in breeding areas for longspurs may make estimation of these vital rates difficult (Sedgwick, 2004; With, 2021). Recent advancements in VHF technology (e.g., Motus Wildlife Tracking System) may allow for expanded assessments of seasonal fecundity and annual survival of both adults and juveniles, in addition to movement rates between habitat types.

Evaluating stress hormone levels sensu Des Brisay (2018) of fledglings, juveniles, and adult longspurs in crop sites could provide additional insight into habitat quality. Body condition at the start of migration often influences survival of adults and juveniles during migration and winter (Angelier et al., 2011; Labocha & Hayes, 2012; Merilä & Svensson, 1997). In Europe, Kuiper et al. (2015) found that cereal fields used by nesting skylarks contained less abundant food resources, resulting in lower nestling weights. Lower post‐fledging survival in crop sites, reduced condition of adults or young, or lower seasonal fecundity in crop sites would provide evidence for reduced habitat quality of crop sites.

Finally, our study area contained large tracts of both crop and native habitat, and we have not assessed the use of croplands by longspurs in areas with less‐extensive native prairie. Use of croplands as nesting habitat may be limited to areas close to historical native prairie habitats, depending on habitat selection behavior at higher spatial scales (e.g., first‐order habitat selection; Johnson, 1980).

### 4.5 Conservation implications and recommendations

Other studies have demonstrated the importance of croplands as nesting habitats for passerines (Best et al., 1995; Donald et al., 2006; Lokemoen & Beiser, 1997). For example, cereal fields are thought to be the single most important extant habitat for skylarks in Europe (Donald & Vickery, 2000). Given that croplands now occupy a vast proportion of arable regions worldwide, we should encourage management of these lands in ways that enhance nesting habitats for passerines. In particular, management strategies that modify the timing or degree of disturbance in cropland may provide direct benefits to ground‐nesting birds. Odderskær et al. (1997) found that artificially creating open areas (7 m^2^) within cereal fields resulted in higher skylark densities and extended nesting seasons. Recent studies in North America have indicated that autumn‐seeded wheat, or “winter wheat”, may be better for nesting waterfowl and passerines than spring‐seeded wheat due to the increased cover winter wheat provides early in the nesting season and elimination of the need for spring tillage (Davis et al., 2020; Devries et al., 2008; Skone et al., 2016). Although not a viable option for wheat cropping in northcentral Montana, shifting to alternative plantings may be an option in other regions (M. Sather, pers. comm.). However, the appeal of winter wheat depends on species preference for vegetation structure. Species like longspurs that prefer short, sparse vegetation are more likely to prefer spring wheat over winter wheat (Martin & Forsyth, 2003), as winter wheat has taller stature during territory establishment. In Europe, skylarks and yellow wagtails (*Motacilla flava*), which also prefer short‐stature vegetation, abandoned autumn‐seeded crops for spring‐seeded crops late in the season as crop height increased, resulting in lost nesting opportunities in autumn‐seeded crops (Eggers et al., 2011; Gilroy et al., 2010).

Although overall nest survival rates were similar in crop and native sites, reproductive output might be improved with modified farming practices that minimize the destruction of early nests. Early seeding (<10 May) and reducing summer fallowing should result in greater use and higher nest survival by longspurs in crop fields. Pesticides, including fungicides, can harm adults and nestlings (Martin et al., 1998; McEwen & Ells, 1975; Mineau & Whiteside, 2013). Thus, we recommend reduction or elimination of widespread application of herbicides, pesticides, and fungicides when possible, until their potential impacts on the survival of nesting longspurs can be evaluated.

## 5 CONCLUSIONS

The loss of native grasslands through conversion to cropland is a primary driver of grassland bird population declines, including thick‐billed longspurs (Blann, 2006; Ellis et al., 2010; Samson et al., 2004; Wright & Wimberly, 2013). Nevertheless, we show that longspurs breeding in a region of relatively intact grasslands in northeastern Montana appear to nest successfully in nearby crop fields. While hazards for nests (e.g., farming activities and predation) differed between crop and native sites, overall seasonal reproductive output and nesting success were similar.

Due to the loss of large‐scale, historic disturbance regimes on native sites, crop fields may effectively expand nesting opportunities in a region where native habitat has been confined to patches with arid soils. Minor modifications to cultivated land management practices in this region could benefit longspurs, particularly in relation to timing of mechanical operations. Appropriate management of cultivated fields for conservation depends entirely on the species and agricultural system, making ubiquitous recommendations across species and systems inappropriate. In addition, such management strategies should be restricted to cultivated fields to preclude deleterious effects on grassland obligate species (Davis et al., 2020). Given the great weight of evidence that conversion to cropland is detrimental to grassland bird populations, we strongly recommend against any conversion of native prairie to benefit longspurs. Additionally, future research should explore management practices that promote dynamic patterns of disturbance, bare ground, and short grass in native prairies, especially in early spring when longspurs select territories. Further investigation into population demographics, body condition, and resource availability may provide additional insight into the relationship between longspurs and cropland.

## AUTHOR CONTRIBUTIONS

**Amber E. Swicegood:** Conceptualization (equal); formal analysis (lead); investigation (lead); methodology (equal); validation (equal); visualization (lead); writing – original draft (lead); writing – review and editing (equal). **Kevin S. Ellison:** Conceptualization (equal); funding acquisition (equal); investigation (equal); methodology (equal); resources (equal); validation (equal); writing – original draft (supporting); writing – review and editing (equal). **Marisa Sather:** Conceptualization (equal); funding acquisition (equal); investigation (supporting); methodology (equal); project administration (equal); resources (equal); validation (equal); writing – original draft (supporting); writing – review and editing (equal). **Scott G. Somershoe:** Conceptualization (equal); funding acquisition (lead); investigation (equal); methodology (supporting); project administration (equal); resources (equal); validation (equal); writing – original draft (supporting); writing – review and editing (equal). **Lance B. McNew:** Conceptualization (lead); formal analysis (supporting); funding acquisition (lead); investigation (equal); methodology (equal); project administration (equal); supervision (lead); validation (equal); visualization (equal); writing – original draft (equal); writing – review and editing (equal).

## CONFLICT OF INTEREST STATEMENT

None declared.

## Supporting information


Appendix S1

Appendix S2

Appendix S3

## Data Availability

Sampling locations, data, and scripts used for analysis are all available at the Dryad Digital Repository: https://doi.org/10.5061/dryad.7sqv9s4x3.

## References

[ece39993-bib-0001] Abrams, P. A. , Ruokolainen, L. , Shuter, B. J. , & McCann, K. S. (2012). Harvesting creates ecological traps: Consequences of invisible mortality risks in predator–prey metacommunities. Ecology, 93, 281–293. 22624310 10.1890/11-0011.1

[ece39993-bib-0002] Angelier, F. , Tonra, C. M. , Holberton, R. L. , & Marra, P. P. (2011). Short‐term changes in body condition in relation to habitat and rainfall abundance in American redstarts *Setophaga ruticilla* during the non‐breeding season. Journal of Avian Biology, 42, 335–341.

[ece39993-bib-0003] Arnold, T. W. (2010). Uninformative parameters and model selection using Akaike's Information Criterion. The Journal of Wildlife Management, 74, 1175–1178.

[ece39993-bib-0004] Badyaev, A. V. , Hill, G. E. , & Beck, M. L. (2003). Interaction between maternal effects: Onset of incubation and offspring sex in two populations of a passerine bird. Oecologia, 135, 386–390. 12721828 10.1007/s00442-003-1203-x

[ece39993-bib-0005] Basore, N. S. , Best, L. B. , & Wooley, J. B., Jr. (1986). Bird nesting in Iowa no‐tillage and tilled cropland. The Journal of Wildlife Management, 50, 19–28.

[ece39993-bib-0006] Battin, J. (2004). When good animals love bad habitats: Ecological traps and the conservation of animal populations. Conservation Biology, 18, 1482–1491.

[ece39993-bib-0007] Best, L. B. , Campa, H., III , Kemp, K. E. , Robel, R. J. , Ryan, M. R. , Savidge, J. A. , Weeks, H. P., Jr. , & Winterstein, S. R. (1997). Bird abundance and nesting in CRP fields and cropland in the Midwest: A regional approach. Wildlife Society Bulletin, 25, 864–877.

[ece39993-bib-0008] Best, L. B. , Freemark, K. E. , Dinsmore, J. J. , & Camp, M. (1995). A review and synthesis of habitat use by breeding birds in agricultural landscapes of Iowa. American Midland Naturalist, 134, 1–29.

[ece39993-bib-0009] Blann, K. (2006). Habitat in agricultural landscapes: How much is enough. A state‐of‐the‐science literature review. Defenders of Wildlife.

[ece39993-bib-0010] Burnham, K. P. , & Anderson, D. R. (2002). Model selection and multimodel inference. Springer.

[ece39993-bib-0011] Burnham, K. P. , Anderson, D. R. , & Huyvaert, K. P. (2011). AIC model selection and multimodel inference in behavioral ecology: Some background, observations, and comparisons. Behavioral Ecology and Sociobiology, 65, 23–35.

[ece39993-bib-0012] Charboneau, J. L. , Nelson, B. , & Hartman, R. L. (2013). A floristic inventory of Phillips and Valley Counties, Montana (USA). Journal of the Botanical Research Institute of Texas, 7, 847–878.

[ece39993-bib-0013] Chen, K. , Cheng, Y. , Berkout, O. , & Lindhiem, O. (2017). Analyzing proportion scores as outcomes for prevention trials: A statistical primer. Prevention Science, 18, 312–321. 26960687 10.1007/s11121-016-0643-6 PMC5860877

[ece39993-bib-0014] Cooper, S. V. , Hendricks, P. , & Jean, C. (2001). Biological survey of a prairie landscape in Montana's glaciated plains. Final Report. *Montana Natural Heritage Survey* . https://mtnhp.org/plants/reports/bittercreek.pdf

[ece39993-bib-0015] Coupland, R. T. (1961). A reconsideration of grassland classification in the northern Great Plains of North America. The Journal of Ecology, 49, 135–167.

[ece39993-bib-0016] Daubenmire, R. F. (1959). Canopy coverage method of vegetation analysis. Northwest Science, 33, 39–64.

[ece39993-bib-0017] Davis, S. K. , Kirk, D. A. , Armstrong, L. M. , Devries, J. H. , & Fisher, R. J. (2020). Shifting from spring wheat to winter wheat: A potential conservation strategy for grassland songbirds in cultivated landscapes? Biological Conservation, 245, 108530.

[ece39993-bib-0018] Delibes, M. , Ferreras, P. , & Gaona, P. (2001). Attractive sinks, or how individual behavioural decisions determine source–sink dynamics. Ecology Letters, 4, 401–403.

[ece39993-bib-0019] Des Brisay, P. G. (2018). Effects of oil development on habitat quality and its perception by mixed‐grass prairie songbirds .

[ece39993-bib-0020] Devries, J. H. , Brook, R. W. , Howerter, D. W. , & Anderson, M. G. (2008). Effects of spring body condition and age on reproduction in mallards (*Anas platyrhynchos*). The Auk, 125, 618–628.

[ece39993-bib-0021] Diefenbach, D. R. , Brauning, D. W. , & Mattice, J. A. (2003). Variability in grassland bird counts related to observer differences and species detection rates. The Auk, 120, 1168–1179.

[ece39993-bib-0022] Donald, P. F. , Sanderson, F. J. , Burfield, I. J. , & Van Bommel, F. P. (2006). Further evidence of continent‐wide impacts of agricultural intensification on European farmland birds, 1990–2000. Agriculture, Ecosystems & Environment, 116, 189–196.

[ece39993-bib-0023] Donald, P. F. , & Vickery, J. A. (2000). The importance of cereal fields to breeding and wintering skylarks (*Alauda arvensis*) in the UK. Ecology and Conservation of Lowland Farmland Birds, 140, 150.

[ece39993-bib-0024] Dunn, P. K. , & Smyth, G. K. (2018). Generalized linear models with examples in R (Vol. 53). Springer.

[ece39993-bib-0025] Eggers, S. , Unell, M. , & Pärt, T. (2011). Autumn‐sowing of cereals reduces breeding bird numbers in a heterogeneous agricultural landscape. Biological Conservation, 144, 1137–1144.

[ece39993-bib-0026] Ellis, E. C. , Klein Goldewijk, K. , Siebert, S. , Lightman, D. , & Ramankutty, N. (2010). Anthropogenic transformation of the biomes, 1700–2000. Global Ecology and Biogeography, 19, 589–606.

[ece39993-bib-0027] ESRI . (2019). Version 10.4.1 . Environmental Systems Research Institute.

[ece39993-bib-0028] Felske, B. E. (1971). Population dynamics and productivity of McCown's longspur at matador, Saskatchewan (Thesis). University of Saskatchewan, Saskatoon, Canada. 144 pp.

[ece39993-bib-0029] Fiske, I. , & Chandler, R. (2011). Unmarked: An R package for fitting hierarchical models of wildlife occurrence and abundance. Journal of Statistical Software, 43, 1–23.

[ece39993-bib-0030] Fletcher, R. J., Jr. , Orrock, J. L. , & Robertson, B. A. (2012). How the type of anthropogenic change alters the consequences of ecological traps. Proceedings of the Royal Society B: Biological Sciences, 279, 2546–2552. 10.1098/rspb.2012.0139 PMC3350704 22378802

[ece39993-bib-0031] Fuhlendorf, S. , & Engle, D. (2004). Application of the fire–grazing interaction to restore a shifting mosaic on tallgrass prairie. Journal of Applied Ecology, 41, 604–614.

[ece39993-bib-0032] Gaudet, C. A. , Green, E. N. , Brigham, R. M. , & Davis, S. K. (2020). Nesting ecology and reproductive success of mixed‐grass prairie songbirds. The Wilson Journal of Ornithology, 132, 952–966.

[ece39993-bib-0033] Gilroy, J. , Anderson, G. , Vickery, J. , Grice, P. , & Sutherland, W. (2011). Identifying mismatches between habitat selection and habitat quality in a ground‐nesting farmland bird. Animal Conservation, 14, 620–629.

[ece39993-bib-0034] Gilroy, J. J. , Anderson, G. Q. , Grice, P. V. , Vickery, J. A. , & Sutherland, W. J. (2010). Mid‐season shifts in the habitat associations of yellow wagtails *Motacilla flava* breeding in arable farmland. Ibis, 152, 90–104.

[ece39993-bib-0035] Gilroy, J. J. , & Sutherland, W. J. (2007). Beyond ecological traps: Perceptual errors and undervalued resources. Trends in Ecology & Evolution, 22, 351–356. 17416438 10.1016/j.tree.2007.03.014

[ece39993-bib-0036] Giovanni, M. D. , Van Der Burg, M. P. , Anderson, L. C. , Powell, L. A. , Schacht, W. H. , & Tyre, A. J. (2011). Estimating nest density when detectability is incomplete: Variation in nest attendance and response to disturbance by western meadowlarks. The Condor, 113, 223–232.

[ece39993-bib-0037] Golding, J. D. , & Dreitz, V. J. (2017). Songbird response to rest‐rotation and season‐long cattle grazing in a grassland sagebrush ecosystem. Journal of Environmental Management, 204, 605–612. 28946000 10.1016/j.jenvman.2017.09.044

[ece39993-bib-0038] Greer, R. D. (1988). Effects of habitat structure and productivity on grassland birds (Ph.D. dissertation). University of Wyoming. 128 p.

[ece39993-bib-0039] Greer, R. D. , & Anderson, S. H. (1989). Relationships between population demography of McCown's longspurs and habitat resources. The Condor, 91, 609–619.

[ece39993-bib-0040] Hale, R. , & Swearer, S. E. (2016). Ecological traps: Current evidence and future directions. Proceedings of the Royal Society B: Biological Sciences, 283, 20152647. 10.1098/rspb.2015.2647 PMC4760169 26865295

[ece39993-bib-0041] Hale, R. , Treml, E. A. , & Swearer, S. E. (2015). Evaluating the metapopulation consequences of ecological traps. Proceedings of the Royal Society B: Biological Sciences, 282, 20142930. 10.1098/rspb.2014.2930 PMC4375870 25761712

[ece39993-bib-0042] Hébert, P. (2002). Ecological factors affecting initiation of incubation behaviour. In D. C. Deeming (Ed.), Avian incubation: Behaviour, environment, and evolution (pp. 271–279). Oxford University Press.

[ece39993-bib-0043] Hovick, T. J. , Elmore, R. D. , Fuhlendorf, S. D. , Engle, D. M. , & Hamilton, R. G. (2015). Spatial heterogeneity increases diversity and stability in grassland bird communities. Ecological Applications, 25, 662–672. 26214912 10.1890/14-1067.1

[ece39993-bib-0044] Igl, L. D. , & Johnson, D. H. (1997). Changes in breeding bird populations in North Dakota: 1967 to 1992–93. The Auk, 114, 74–92.

[ece39993-bib-0045] Johnson, D. H. (1980). The comparison of usage and availability measurements for evaluating resource preference. Ecology, 61, 65–71.

[ece39993-bib-0046] Jones, S. L. , Dieni, J. S. , & Gouse, P. J. (2010). Reproductive biology of a grassland songbird community in northcentral Montana. The Wilson Journal of Ornithology, 122, 455–464.

[ece39993-bib-0047] Jongsomjit, D. , Jones, S. L. , Gardali, T. , Geupel, G. R. , & Gouse, P. J. (2007). A guide to nestling development and aging in altricial passerines .

[ece39993-bib-0048] Kéry, M. , & Chandler, R. (2016). Dynamic occupancy models in unmarked . https://mran.microsoft.com/snapshot/2017‐06‐27/web/packages/unmarked/vignettes/colext.pdf

[ece39993-bib-0049] Kladivko, E. J. (2001). Tillage systems and soil ecology. Soil and Tillage Research, 61, 61–76.

[ece39993-bib-0050] Klett, A. T. , Duebbert, H. F. , Faanes, C. A. , & Higgins, K. F. (1986). Techniques for studying nest success of ducks in upland habitats in the prairie pothole region. U.S. Fish & Wildlife Service.

[ece39993-bib-0051] Knapp, A. K. , Blair, J. M. , Briggs, J. M. , Collins, S. L. , Hartnett, D. C. , Johnson, L. C. , & Towne, E. G. (1999). The keystone role of bison in north American tallgrass prairie: Bison increase habitat heterogeneity and alter a broad array of plant, community, and ecosystem processes. Bioscience, 49, 39–50.

[ece39993-bib-0052] Knopf, F. L. (1996). Prairie legacies‐birds. In F. B. Samson & F. L. Knopf (Eds.), Prairie conservation: Preserving North America's most endangered ecosystem (pp. 135–148). Island Press.

[ece39993-bib-0053] Koford, R. R. (1999). Density and fledging success of grassland birds in conservation reserve program fields in North Dakota and west‐Central Minnesota. Studies in Avian Biology, 19, 187–195.

[ece39993-bib-0054] Kuiper, M. , Ottens, H. , Van Ruijven, J. , Koks, B. , de Snoo, G. , & Berendse, F. (2015). Effects of breeding habitat and field margins on the reproductive performance of skylarks (*Alauda arvensis*) on intensive farmland. Journal of Ornithology, 156, 557–568.

[ece39993-bib-0055] Laake, J. L. (2013). RMark: An R interface for analysis of capture‐recapture data with MARK . AFSC Processed Rep 2013‐01, 25p. Alaska Fisheries Science Cent., NOAA, Natl. Mar. Fish. Serv., 7600 Sand Point Way NE, Seattle WA 98115.

[ece39993-bib-0056] Labocha, M. K. , & Hayes, J. P. (2012). Morphometric indices of body condition in birds: A review. Journal of Ornithology, 153, 1–22.

[ece39993-bib-0057] Lenard, S. , Carlson, J. , Hendricks, P. , & Currier, C. (2006). Grassland bird surveys in north Valley County, Montana: Progress report. Report to the Bureau of Land Management. Montana Natural Heritage Program.

[ece39993-bib-0058] Lipsey, M. K. S. (2015). Cows and plows: Science‐based conservation for grassland songbirds in agricultural landscapes (Ph.D. dissertation). University of Montana, Missoula, MT. 137 pp.

[ece39993-bib-0059] Lloyd, J. D. , & Martin, T. E. (2005). Reproductive success of chestnut‐collared longspurs in native and exotic grassland. The Condor, 107, 363–374.

[ece39993-bib-0060] Lokemoen, J. T. , & Beiser, J. A. (1997). Bird use and nesting in conventional, minimum‐tillage, and organic cropland. The Journal of Wildlife Management, 61, 644–655.

[ece39993-bib-0061] Long, J. A. , Lawrence, R. L. , Miller, P. R. , Marshall, L. A. , & Greenwood, M. C. (2014). Adoption of cropping sequences in Northeast Montana: A spatio‐temporal analysis. Agriculture, Ecosystems & Environment, 197, 77–87.

[ece39993-bib-0062] Loss, S. R. , Will, T. , & Marra, P. P. (2015). Direct mortality of birds from anthropogenic causes. Annual Review of Ecology, Evolution, and Systematics, 46, 99–120.

[ece39993-bib-0063] Mackenzie, D. I. (2006). Modeling the probability of resource use: The effect of, and dealing with, detecting a species imperfectly. The Journal of Wildlife Management, 70, 367–374.

[ece39993-bib-0064] MacKenzie, D. I. , Nichols, J. D. , Hines, J. E. , Knutson, M. G. , & Franklin, A. B. (2003). Estimating site occupancy, colonization, and local extinction when a species is detected imperfectly. Ecology, 84, 2200–2207.

[ece39993-bib-0065] Mahoney, A. , & Chalfoun, A. D. (2016). Reproductive success of horned lark and McCown's longspur in relation to wind energy infrastructure. The Condor: Ornithological Applications, 118, 360–375.

[ece39993-bib-0066] Martin, P. A. , & Forsyth, D. J. (2003). Occurrence and productivity of songbirds in prairie farmland under conventional versus minimum tillage regimes. Agriculture, Ecosystems & Environment, 96, 107–117.

[ece39993-bib-0067] Martin, P. A. , Johnson, D. L. , Forsyth, D. J. , & Hill, B. D. (1998). Indirect effects of the pyrethroid insecticide deltamethrin on reproductive success of chestnut‐collared longspurs. Ecotoxicology, 7, 89–97.

[ece39993-bib-0068] Martin, T. E. , & Geupel, G. R. (1993). Nest‐monitoring plots: Methods for locating nests and monitoring success (Métodos Para localizar nidos y monitorear el éxito de estos). Journal of Field Ornithology, 64, 507–519.

[ece39993-bib-0069] Mazerolle, M. J. (2020). AICcmodavg: Model selection and multimodel inference based on (Q)AIC(c) . R Package version 2.3‐1. https://cran.r‐project.org/package=AICcmodavg

[ece39993-bib-0070] McEwen, L. C. , & Ells, J. O. (1975). Field ecology investigations of the effects of selected pesticides on wildlife populations. Colorado State University.

[ece39993-bib-0071] Merilä, J. , & Svensson, E. (1997). Are fat reserves in migratory birds affected by condition in early life? Journal of Avian Biology, 28, 279–286.

[ece39993-bib-0072] Mickey, F. W. (1943). Breeding habits of McCown's longspur. The Auk, 60, 181–209.

[ece39993-bib-0073] Miller, P. R. , McConkey, B. G. , Clayton, G. W. , Brandt, S. A. , Staricka, J. A. , Johnston, A. M. , Lafond, G. P. , Schatz, B. G. , Baltensperger, D. D. , & Neill, K. E. (2002). Pulse crop adaptation in the northern Great Plains. Agronomy Journal, 94, 261–272.

[ece39993-bib-0074] Mineau, P. , & Whiteside, M. (2013). Pesticide acute toxicity is a better correlate of US grassland bird declines than agricultural intensification. PLoS One, 8, e57457. 23437392 10.1371/journal.pone.0057457 PMC3577736

[ece39993-bib-0075] Montgomery, D. C. , Peck, E. A. , & Vining, G. G. (2021). Introduction to linear regression analysis (6th ed.). John Wiley & Sons.

[ece39993-bib-0076] Odderskær, P. , Prang, A. , Poulsen, J. G. , Andersen, P. N. , & Elmegaard, N. (1997). Skylark (*Alauda arvensis*) utilisation of micro‐habitats in spring barley fields. Agriculture, Ecosystems & Environment, 62, 21–29.

[ece39993-bib-0077] Pagano, A. M. , & Arnold, T. W. (2009). Detection probabilities for ground‐based breeding waterfowl surveys. The Journal of Wildlife Management, 73, 392–398.

[ece39993-bib-0078] Perlut, N. G. , Strong, A. M. , Donovan, T. M. , & Buckley, N. J. (2008). Regional population viability of grassland songbirds: Effects of agricultural management. Biological Conservation, 141, 3139–3151.

[ece39993-bib-0079] Pietz, P. J. , & Granfors, D. A. (2000). Identifying predators and fates of grassland passerine nests using miniature video cameras. The Journal of Wildlife Management, 64, 71–87.

[ece39993-bib-0080] Pietz, P. J. , Granfors, D. A. , Ribic, C. A. , & Thompson, F. (2012). Knowledge gained from video‐monitoring grassland passerine nests. Video Surveillance of Nesting Birds, 43, 3–22.

[ece39993-bib-0081] Pimentel, D. , Harvey, C. , Resosudarmo, P. , Sinclair, K. , Kurz, D. , McNair, M. , Crist, S. , Shpritz, L. , Fitton, L. , & Saffouri, R. (1995). Environmental and economic costs of soil erosion and conservation benefits. Science, 267, 1117–1123. 17789193 10.1126/science.267.5201.1117

[ece39993-bib-0082] Powell, L. A. (2007). Approximating variance of demographic parameters using the delta method: A reference for avian biologists. The Condor, 109, 949–954.

[ece39993-bib-0083] PRISM Climate Group, O. S. U . (2022). http://prism.oregonstate.edu

[ece39993-bib-0084] Pulliam, H. R. (1988). Sources, sinks, and population regulation. The American Naturalist, 132, 652–661.

[ece39993-bib-0085] Pulliam, J. P. , Somershoe, S. , Sather, M. , & McNew, L. B. (2021). Nest density drives productivity in chestnut‐collared longspurs: Implications for grassland bird conservation. PLoS One, 16, e0256346. 34428226 10.1371/journal.pone.0256346 PMC8384174

[ece39993-bib-0086] Quinn, J. E. , Awada, T. , Trindade, F. , Fulginiti, L. , & Perrin, R. (2017). Combining habitat loss and agricultural intensification improves our understanding of drivers of change in avian abundance in a North American cropland anthrome. Ecology and Evolution, 7, 803–814.

[ece39993-bib-0087] R Core Team . (2021). R: A language and environment for statistical computing. R Foundation for Statistical Computing.

[ece39993-bib-0088] Ralph, C. J. (1993). Handbook of field methods for monitoring landbirds (Vol. 144). Pacific Southwest Research Station.

[ece39993-bib-0089] Reintsma, K. M. , Delamont, M. M. , Berkeley, L. I. , & Dreitz, V. J. (2022). Thick‐billed longspur (*Rhynchophanes mccownii*) reproduction shows minimal short‐term response to conservation‐based program. The Wilson Journal of Ornithology, 134(2), 365–372.

[ece39993-bib-0090] Ribic, C. A. , Guzy, M. J. , Anderson, T. J. , Sample, D. W. , & Nack, J. L. (2012). Bird productivity and nest predation in agricultural grasslands . USGS Northern Prairie Wildlife Research Center. 257. https://digitalcommons.unl.edu/usgsnpwrc/257

[ece39993-bib-0091] Robel, R. , Briggs, J. , Dayton, A. , & Hulbert, L. (1970). Relationships between visual obstruction measurements and weight of grassland vegetation. Rangeland Ecology & Management/Journal of Range Management Archives, 23, 295–297.

[ece39993-bib-0092] Robertson, B. A. , & Hutto, R. L. (2006). A framework for understanding ecological traps and an evaluation of existing evidence. Ecology, 87, 1075–1085. 16761584 10.1890/0012-9658(2006)87[1075:affuet]2.0.co;2

[ece39993-bib-0093] Robertson, B. A. , Rehage, J. S. , & Sih, A. (2013). Ecological novelty and the emergence of evolutionary traps. Trends in Ecology & Evolution, 28, 552–560. 23756104 10.1016/j.tree.2013.04.004

[ece39993-bib-0094] Rosenberg, K. V. , Dokter, A. M. , Blancher, P. J. , Sauer, J. R. , Smith, A. C. , Smith, P. A. , Stanton, J. C. , Panjabi, A. , Helft, L. , & Parr, M. (2019). Decline of the north American avifauna. Science, 366, 120–124. 31604313 10.1126/science.aaw1313

[ece39993-bib-0095] Rotella, J. J. , Dinsmore, S. J. , & Shaffer, T. L. (2004). Modeling nest‐survival data: A comparison of recently developed methods that can be implemented in MARK and SAS. Animal Biodiversity and Conservation, 27, 187–205.

[ece39993-bib-0096] Royle, J. A. , Dawson, D. K. , & Bates, S. (2004). Modeling abundance effects in distance sampling. Ecology, 85, 1591–1597.

[ece39993-bib-0097] Samson, F. , & Knopf, F. (1994). Prairie conservation in North America. Bioscience, 44, 418–421.

[ece39993-bib-0098] Samson, F. B. , & Knopf, F. L. (1996). Prairie conservation: Preserving North America's most endangered ecosystem. Island Press.

[ece39993-bib-0099] Samson, F. B. , Knopf, F. L. , & Ostlie, W. R. (2004). Great plains ecosystems: Past, present, and future. Wildlife Society Bulletin, 32, 6–15.

[ece39993-bib-0100] Santangeli, A. , Lehikoinen, A. , Bock, A. , Peltonen‐Sainio, P. , Jauhiainen, L. , Girardello, M. , & Valkama, J. (2018). Stronger response of farmland birds than farmers to climate change leads to the emergence of an ecological trap. Biological Conservation, 217, 166–172.

[ece39993-bib-0101] Sauer, J. R. , Link, W. A. , & Hines, J. E. (2020). The North American Breeding Bird Survey, results and analysis 1966–2019. U.S. Geological Survey Data Release. 10.5066/P96A7675

[ece39993-bib-0102] Schlaepfer, M. A. , Runge, M. C. , & Sherman, P. W. (2002). Ecological and evolutionary traps. Trends in Ecology & Evolution, 17, 474–480.

[ece39993-bib-0103] Sedgwick, J. (2004). McCown's longspur (Rhynchophanes mccownii): A technical conservation assessment. USDA Forest Service, Rocky Mountain Region.

[ece39993-bib-0104] Shaffer, J. A. , Igl, L. D. , Johnson, D. H. , Sondreal, M. L. , Goldade, C. M. , Rabie, P. A. , Wooten, T. L. , & Euliss, B. R. (2019). The effects of management practices on grassland birds—Thick‐billed longspur (Rhynchophanes mccownii) . Report 1842Y.

[ece39993-bib-0105] Simon, R. N. , & Fortin, D. (2019). Linking habitat use to mortality and population viability to disarm an ecological trap. Biological Conservation, 236, 366–374.

[ece39993-bib-0106] Skagen, S. K. , Augustine, D. J. , & Derner, J. D. (2018). Semi‐arid grassland bird responses to patch‐burn grazing and drought. The Journal of Wildlife Management, 82, 445–456.

[ece39993-bib-0107] Skone, B. R. , Rotella, J. J. , & Walker, J. (2016). Waterfowl production from winter wheat fields in north and South Dakota. The Journal of Wildlife Management, 80, 127–137.

[ece39993-bib-0108] Sollmann, R. , Gardner, B. , Chandler, R. B. , Royle, J. A. , & Sillett, T. S. (2015). An open‐population hierarchical distance sampling model. Ecology, 96, 325–331. 26240853 10.1890/14-1625.1

[ece39993-bib-0109] Somershoe, S. (2018). A full annual‐cycle conservation strategy for Sprague's pipit, chestnut‐collared and McCown's longspurs, and Baird's sparrow. U.S. Department of the Interior, Fish and Wildlife Service.

[ece39993-bib-0110] Song, Y. , Zhou, D. , Zhang, H. , Li, G. , Jin, Y. , & Li, Q. (2013). Effects of vegetation height and density on soil temperature variations. Chinese Science Bulletin, 58, 907–912.

[ece39993-bib-0111] Stinner, B. R. , & House, G. (1990). Arthropods and other invertebrates in conservation‐tillage agriculture. Annual Review of Entomology, 35, 299–318.

[ece39993-bib-0112] Sullivan, B. L. , Wood, C. L. , Iliff, M. J. , Bonney, R. E. , Fink, D. , & Kelling, S. (2020). eBird: A citizen‐based bird observation network in the biological sciences. Biological Conservation, 142, 2282–2292.

[ece39993-bib-0113] Swicegood, A. E. (2022). Fatal attraction for an imperiled songbird: Is cropland in the northern Great Plains an ecological trap for breeding thick‐billed longspur? (M.Sc. thesis). Montana State University. 86 pp.

[ece39993-bib-0114] Van Horne, B. (1983). Density as a misleading indicator of habitat quality. The Journal of Wildlife Management, 47, 893–901.

[ece39993-bib-0115] Van Pelt, R. S. , Hushmurodov, S. X. , Baumhardt, R. L. , Chappell, A. , Nearing, M. A. , Polyakov, V. O. , & Strack, J. E. (2017). The reduction of partitioned wind and water erosion by conservation agriculture. Catena, 148, 160–167.

[ece39993-bib-0116] Vickery, P. D. , Hunter, M. L., Jr. , & Wells, J. V. (1992). Is density an indicator of breeding success? The Auk, 109, 706–710.

[ece39993-bib-0117] Weintraub, K. , George, T. L. , & Dinsmore, S. J. (2016). Nest survival of tricolored blackbirds in California's Central Valley. The Condor: Ornithological Applications, 118, 850–861.

[ece39993-bib-0118] White, G. C. , & Burnham, K. P. (1999). Program MARK: Survival estimation from populations of marked animals. Bird Study, 46, S120–S139.

[ece39993-bib-0119] White, R. P. , Murray, S. , Rohweder, M. , Prince, S. , & Thompson, K. (2000). Grassland ecosystems. World Resources Institute.

[ece39993-bib-0120] Wilson, J. D. , Whittingham, M. J. , & Bradbury, R. B. (2005). The management of crop structure: A general approach to reversing the impacts of agricultural intensification on birds? Ibis, 147, 453–463.

[ece39993-bib-0121] Winter, M. , Hawks, S. E. , Shaffer, J. A. , & Johnson, D. H. (2003). Guidelines for finding nests of passerine birds in tallgrass prairie . USGS Northern Prairie Wildlife Research Center: 160.

[ece39993-bib-0122] With, K. A. (1994). The hazards of nesting near shrubs for a grassland bird, the McCown's longspur. The Condor, 96, 1009–1019.

[ece39993-bib-0123] With, K. A. (2021). Thick‐billed Longspur (*Rhynchophanes mccownii*), version 1.1. In A. F. Poole (Ed.), Birds of the world. Cornell Lab of Ornithology. 10.2173/bow.mcclon.01.1

[ece39993-bib-0124] Wright, C. K. , & Wimberly, M. C. (2013). Recent land use change in the Western Corn Belt threatens grasslands and wetlands. Proceedings of the National Academy of Sciences, 110, 4134–4139. 10.1073/pnas.1215404110 PMC3593829 23431143

