# Supplementary material for: Detecting ecological traps in human‐altered landscapes: A case study of the thick‐billed longspur nesting in croplands
Source: Ecol Evol. 2023 Apr 18;13(4):e9993. doi: 10.1002/ece3.9993 (PMC10111173; doi:10.1002/ece3.9993)
Supplement: Supplementary file 1 — Appendix S1 [file ECE3-13-e9993-s003.zip › ece39993-sup-0001-AppendixS1.docx]

**Appendix A: Initial Surveys to Identify Plots used by Longspurs**

*Site selection.* – Each year we used a restricted random sampling approach to generate 100, 64-ha sampling plots in both crop and native sites. We limited selection to land parcels where we secured permission to conduct fieldwork and to areas that were <3.25 km from accessible roads. Inaccessible areas would have presented a logistical challenge making it difficult to complete surveys in a timely manner. In native sites, we further limited selection to areas with rangeland productivity ≤1,100 kg ha^-1^ to eliminate wetter areas longspurs are known to avoid (Lipsey and Naugle, 2017). Plots were separated by ≥200 meters to ensure independence. From these 100 plots, we randomly selected 50 plots in each site type (crop, native) to survey during the early breeding season (Figure A1). We scouted plots prior to surveys and discarded those that contained water bodies covering ≥1/4 of the plot; in native sites, we also discarded plots that fell within badlands (i.e., canyons, ravines, or gullies consisting of clay-rich soil and extensively eroded by water, precluding the establishment of grassland vegetation) because longspurs do not nest in such areas (DuBois, 1937; Felske, 1971; With and Webb, 1993).

*Initial Occurrence Surveys.* – We conducted initial surveys within the 64-ha plots during 30 Apr–11 May, 2020–21 and completed as many surveys as possible (out of the 50 plots in each habitat type) during the two-week window. Observers walked a U-shaped line transect within each plot, starting 200 meters inward from a randomly selected plot corner (Figure A2; modified from Igl and Johnson, 1997; Golding and Dreitz, 2017). We identified all avian species and recorded perpendicular distance and direction from the transect line for each bird or group of birds seen or heard to maintain consistency with distance sampling methods. Estimated distances were recorded in bins: 0–25, 26–50, 51–75, 76–100, and 101–200 meters. We walked at a pace of 2–3 km hr^-1^ and completed each transect within 40 minutes. Observers were trained to avoid double-counting birds. Surveys began one half hour before sunrise and were completed by 10:00 hours. We did not conduct surveys if wind speed was >25 km hr^-1^ or it was raining. We recorded survey covariates including observer, cloud cover, temperature, wind speed, date, GPS starting point, and transect start/end times. We then used plots that were occupied by thick-billed longspurs to delineate areas for subsequent sampling; we generated smaller, 16-ha plots within occupied areas and used these plots for abundance surveys and nest searching during the rest of the breeding season.

*Initial Occurrence Results.* – In 2020, we conducted initial occurrence surveys in 80 plots (36 crop and 44 native); 67% of crop and 20% of native plots were occupied. In 2021, we conducted initial surveys in 62 plots (35 crop and 27 native); 91% of crop and 33% of native plots were occupied.

FIGURE CAPTIONS

Figure A1. Map of study area and 64-ha plots used for initial surveys on crop and native habitat sites in Valley County, Montana, 2020–21. We generated 50 plots in each site type each year, but not all were surveyed in the two-week survey window. Observers were randomly assigned a few plots to survey each morning. Occupied plots were used to delineate areas for subsequent sampling and nest searching.

Figure A2. Conceptualization of layout of initial survey transects in Valley County, Montana, 2020–21. The dashed lines indicate the transect walked and the outer edge represents the 64-ha survey plot. Detected birds were recorded along with perpendicular distance and direction from the transect line.

REFERENCES CITED

DuBois, A. D. 1937. The McCown Longspurs of a Montana Prairie. The Condor 39:233-238.

Felske, B. E. 1971. Population dynamics and Productivity of McCown's Longspur at Matador, Saskatchewan. Thesis. University of Saskatchewan, Saskatoon, Canada. 144 pages.

Golding, J. D., and V. J. Dreitz. 2017. Songbird response to rest-rotation and season-long cattle grazing in a grassland sagebrush ecosystem. Journal of environmental management 204:605-612.

Igl, L. D., and D. H. Johnson. 1997. Changes in breeding bird populations in North Dakota: 1967 to 1992-93. The Auk 114:74-92.

Lipsey, M. K., and D. E. Naugle. 2017. Precipitation and soil productivity explain effects of grazing on grassland songbirds. Rangeland Ecology & Management 70:331-340.

With, K. A., and D. Webb. 1993. Microclimate of ground nests: the relative importance of radiative cover and wind breaks for three grassland species. The Condor 95:401-413.
