# Supplementary figures and images for: Detecting ecological traps in human‐altered landscapes: A case study of the thick‐billed longspur nesting in croplands
Source: Ecol Evol. 2023 Apr 18;13(4):e9993. doi: 10.1002/ece3.9993 (PMC10111173; doi:10.1002/ece3.9993)

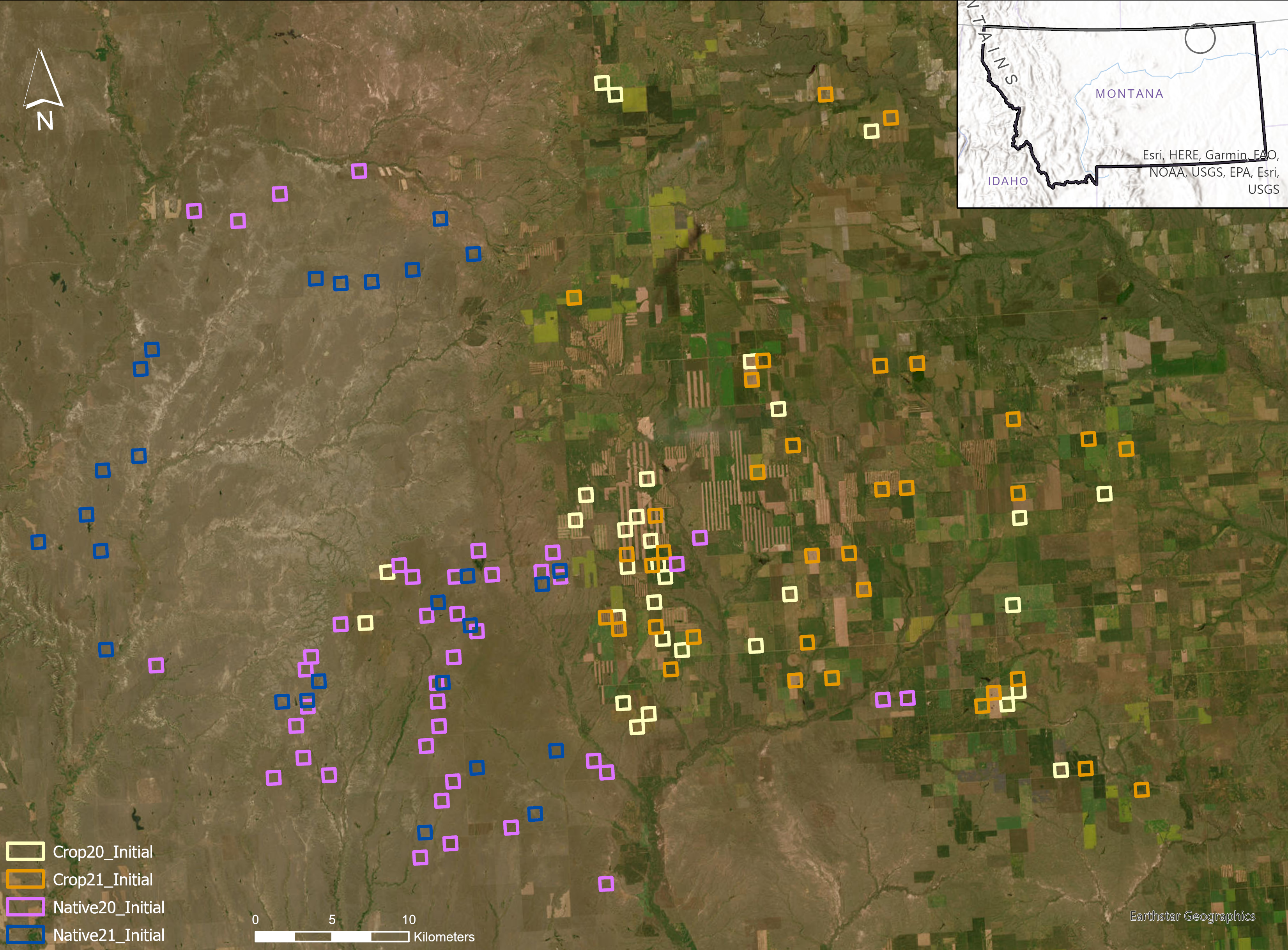

Supplement: Supplementary file 1 — Appendix S1 [file ECE3-13-e9993-s003.zip › ece39993-sup-0003-FigureA1.png]

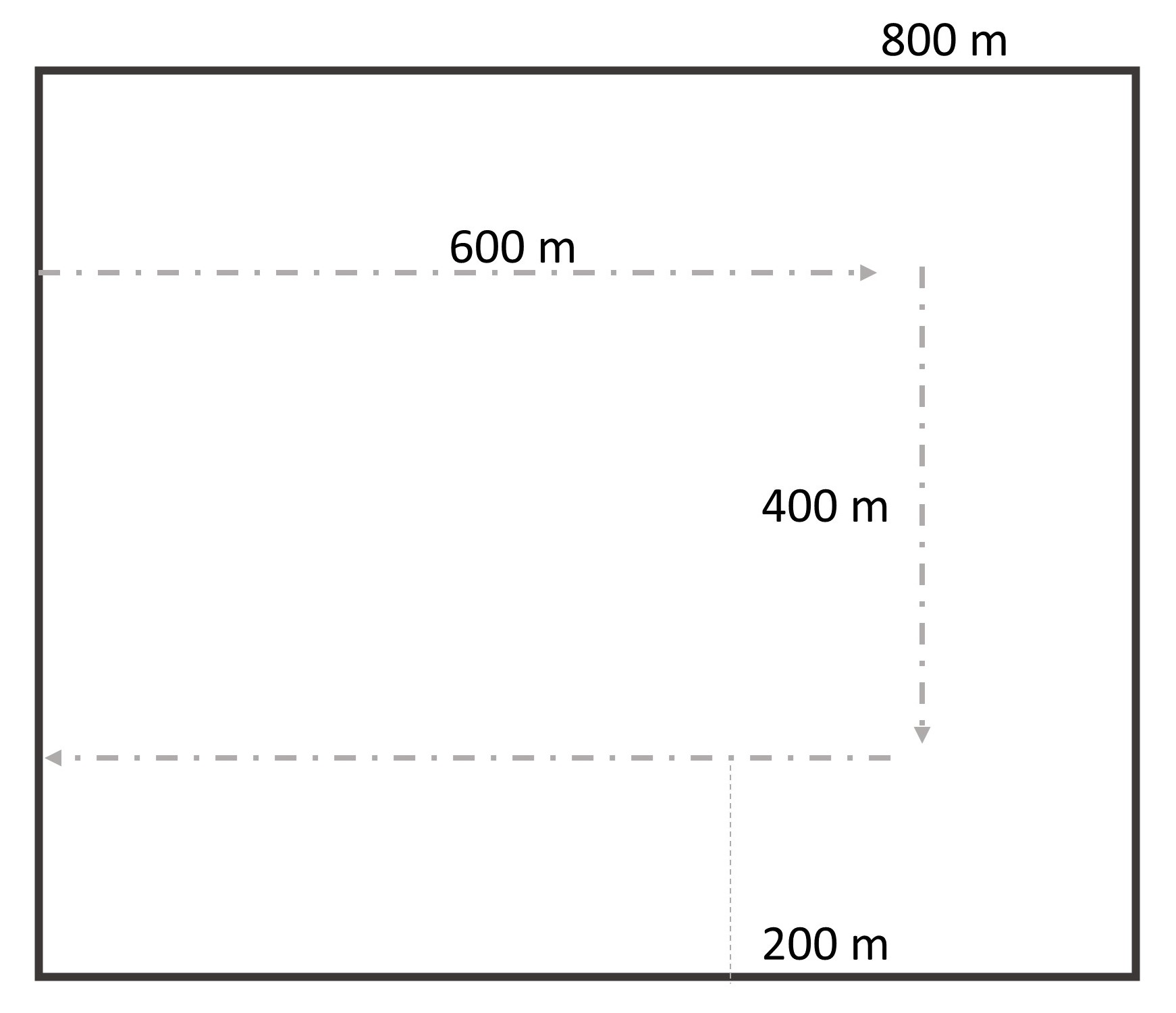

Supplement: Supplementary file 1 — Appendix S1 [file ECE3-13-e9993-s003.zip › ece39993-sup-0004-FigureA2.png]

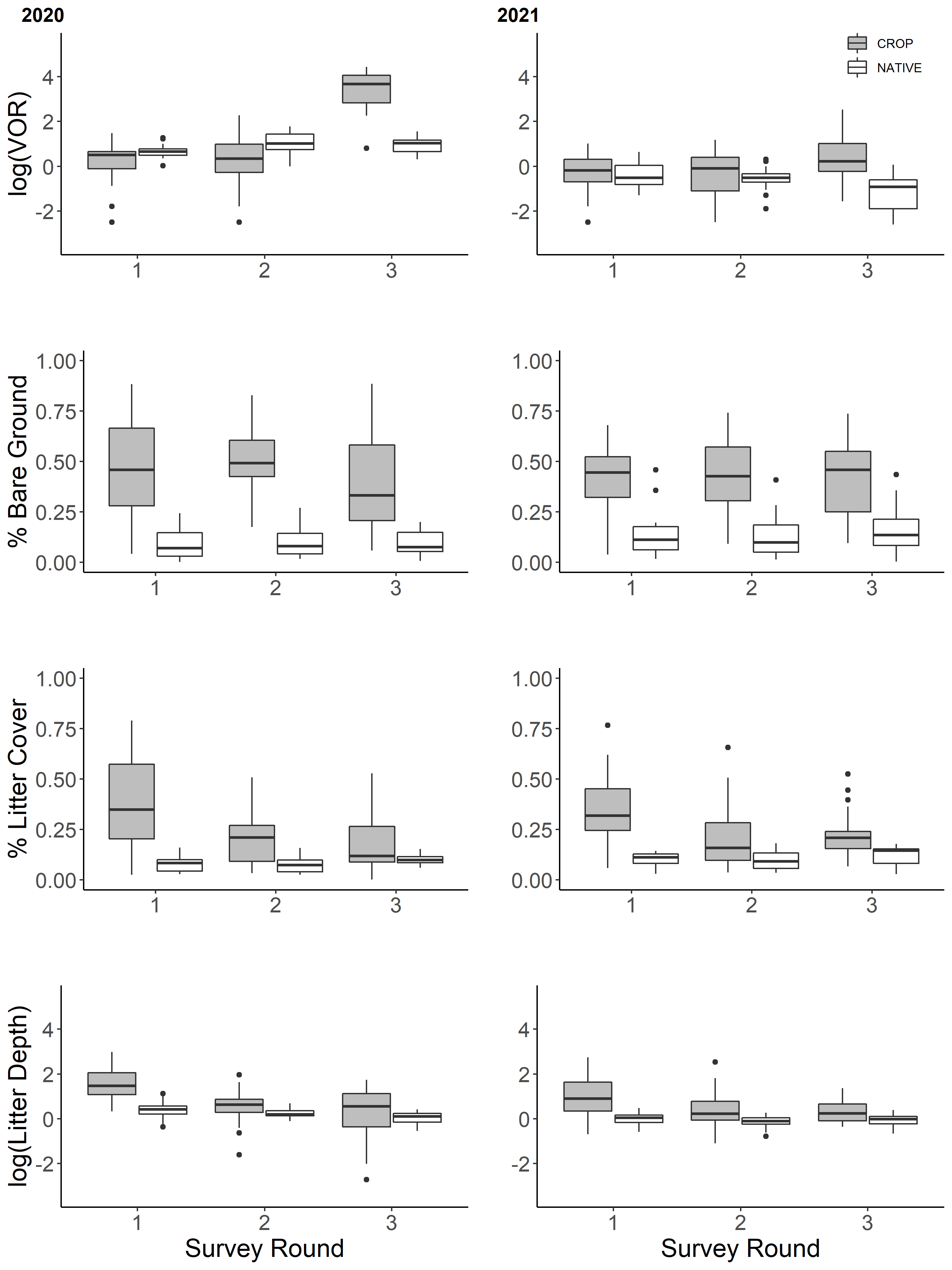

Supplement: Supplementary file 2 — Appendix S2 [file ECE3-13-e9993-s001.zip › ece39993-sup-0005-FigureB1.png]

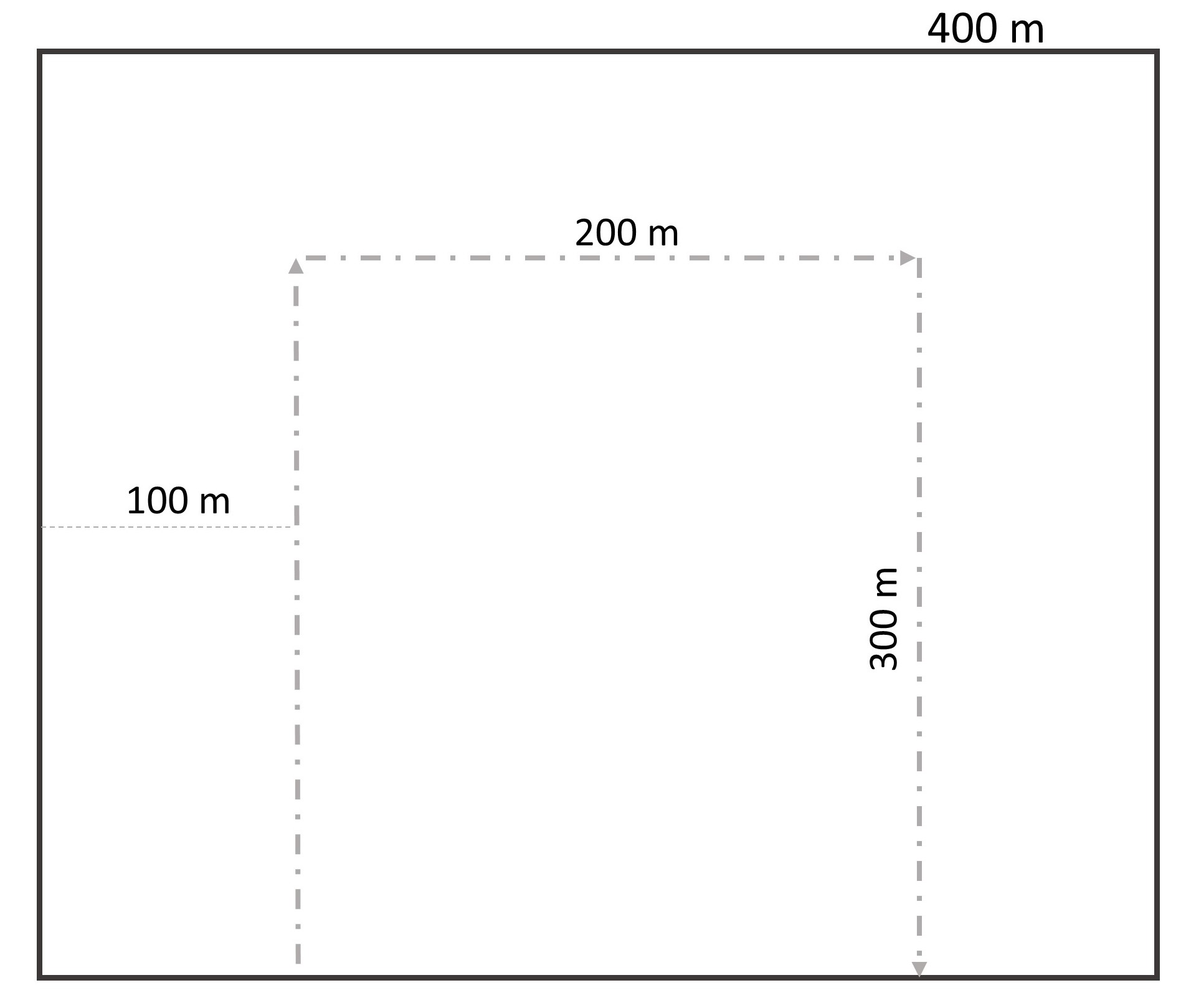

Supplement: Supplementary file 3 — Appendix S3 [file ECE3-13-e9993-s002.zip › ece39993-sup-0006-FigureS1.png]

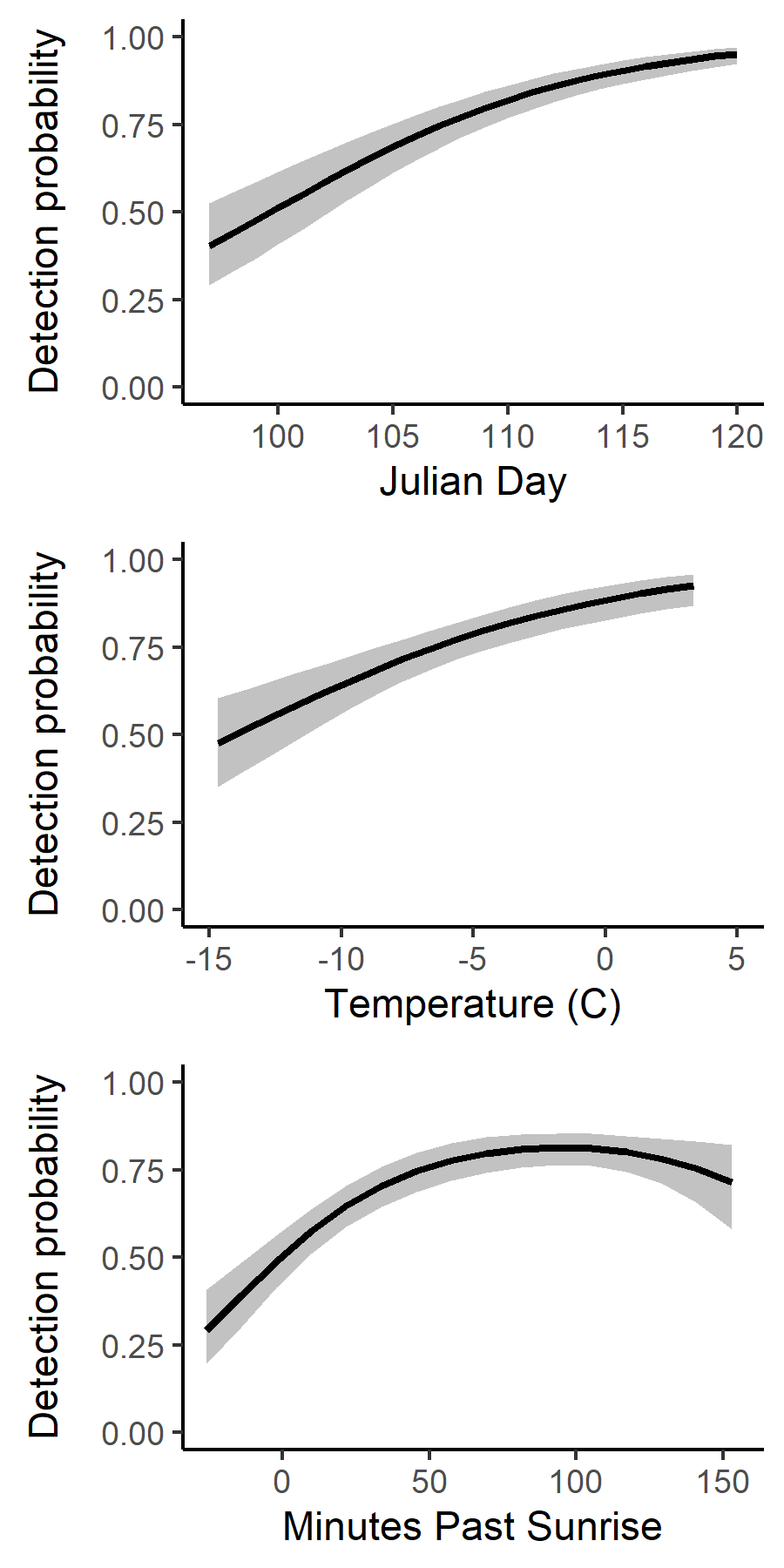

Supplement: Supplementary file 3 — Appendix S3 [file ECE3-13-e9993-s002.zip › ece39993-sup-0007-FigureS2.png]

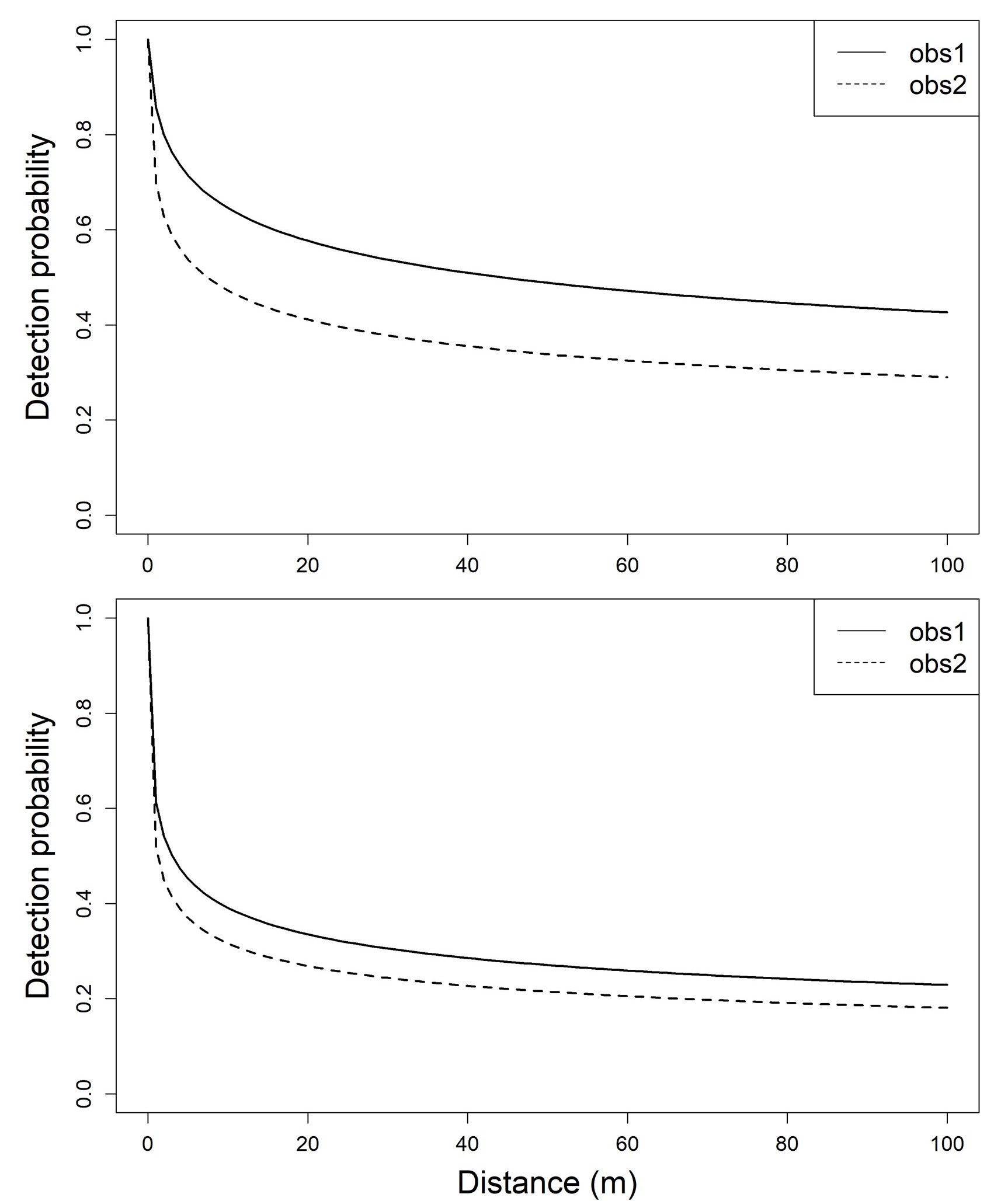

Supplement: Supplementary file 3 — Appendix S3 [file ECE3-13-e9993-s002.zip › ece39993-sup-0008-FigureS3.png]
