# Supplementary material for: Detecting ecological traps in human‐altered landscapes: A case study of the thick‐billed longspur nesting in croplands
Source: Ecol Evol. 2023 Apr 18;13(4):e9993. doi: 10.1002/ece3.9993 (PMC10111173; doi:10.1002/ece3.9993)
Supplement: Supplementary file 2 — Appendix S2 [file ECE3-13-e9993-s001.zip › ece39993-sup-0002-AppendixS2.docx]

**Appendix B: Habitat Condition Results**

In 2020, we surveyed 25 plots in crop sites and 22 plots in native sites 3 times, for a total of 75 and 66 vegetation surveys in crop and native sites, respectively. We completed a total of 225 individual survey points in crop sites and 660 survey points in native sites. In 2021, we surveyed 27 plots in crop sites and 28 plots in native sites 3 times, for a total of 81 and 84 vegetation surveys in crop and native sites, respectively. We completed a total of 243 survey points in crop sites and 840 survey points in native sites (Figure B1).

Visual obstruction reading (VOR) was strongly influenced by the interaction between habitat type and survey round; a model that included this interaction had virtually all support in both years of the study (AIC_c_ *w_i_* = 0.99; Table B1). In 2020, we found evidence that VOR was significantly higher in crop sites during survey round 3 than in survey round 1 (ꞵ = 3.09 ± 0.51) and was lower in native sites than crop sites during the third round (ꞵ = -2.80 ± 0.75). The change in crop VOR by round 3 during 2021 had lower magnitude (ꞵ = 0.72 ± 0.42) but was still lower in native sites than crop sites by round 3 (ꞵ = -1.60 ± 0.59). Bare ground coverage was strongly influenced by the effect of habitat type. The top model, with full support, included the effect of habitat type in both 2020 (AIC_c_ *w_i_* = 0.85) and 2021 (AIC_c_ *w_i_* = 0.88; Table B1). Bare ground was significantly lower on native sites than crop sites during 2020 (ꞵ = -2.03 ± 0.49SE) and 2021 (ꞵ = -1.51 ± 0.39).

The top two models for litter coverage in 2020 included effects of habitat type and survey round and together had the majority of support (AIC_c_ *w_i_* = 0.84; Table B1). However, confidence intervals for the effect size of survey round overlapped zero and this parameter was considered uninformative. The top model for 2021 contained an effect of habitat type (AIC_c_ *w_i_* = 0.66; Table B1). Litter coverage was lower in native sites than crop sites in 2020 (ꞵ = -1.31 ± 0.52) and 2021 (ꞵ = -1.10 ± 0.44). The top model for litter depth both years included the interaction of habitat and survey round. This interaction model carried the majority of support in 2020 (AIC_c_ *w_i_* = 0.84) and in 2021 (AIC_c_ *w_i_* = 0.94; Table B1). In 2020, we found evidence that litter depth was initially lower in native sites than crop sites (ꞵ = -1.08 ± 0.39) and decreased significantly in crop fields as the season progressed (round 2: ꞵ = -0.99 ± 0.37; round 3: ꞵ = -2.09 ± 0.37). In 2021, this variable followed a similar pattern. We found evidence that litter depth was initially lower in native sites than crop sites (ꞵ = -1.00 ± 0.15) and decreased in crop fields as the season progressed (round 2: ꞵ = -0.67 ± 0.15; round 3: ꞵ = -0.71 ± 0.15). For models of residual, forb, and grass cover, the null model was the best supported model in all analyses over both years (ΔAIC_c_ ≤2; Table B1) indicating these vegetation conditions were similar across habitat types and survey rounds.

TABLES

Table B1. Model selection results from generalized linear models predicting differences in specific vegetation conditions in habitats used by thick-billed longspurs in Valley County, Montana, 2020–21^a^. Included are effects of habitat type (*habitat*; crop or native) and survey round (*round*; 3 rounds per season). The number of parameters (K), AIC_c_ values, ΔAIC_c_ values, model weights (*w­_i_*), and cumulative model weights (Cum *w­_i_*) are reported.

^a^Results are based on 225 and 243 survey points in crop sites in 2020 and 2021, respectively, and 660 and 840 survey points in native sites in 2020 and 2021, respectively.

FIGURE CAPTIONS

Figure B1. Visualization of data for four habitat measures collected in crop and native sites in Valley County, Montana, 2020–21. These boxplots illuminate differences in habitat conditions between crop and native sites and changes in conditions over the growing season (May, June, and July, survey rounds 1–3). Measured variables include visual obstruction reading (VOR; top), percent bare ground cover (second), percent litter cover (third), and litter depth (bottom). VOR and litter depth were log-transformed to meet assumptions of linear regression. Plots depict the median (center line), interquartile range (boxes), minimum and maximum (whiskers), and outliers (individual dots).
