# Supplementary material for: Detecting ecological traps in human‐altered landscapes: A case study of the thick‐billed longspur nesting in croplands
Source: Ecol Evol. 2023 Apr 18;13(4):e9993. doi: 10.1002/ece3.9993 (PMC10111173; doi:10.1002/ece3.9993)
Supplement: Supplementary file 3 — Appendix S3 [file ECE3-13-e9993-s002.zip › ece39993-sup-0009-Supinfo.docx]

**Supplementary**

TABLES

Table S1. Breakdown of crop types in which nest searching was conducted for thick-billed longspur in Valley County, Montana 2020–21. Shown are the number of plots in each crop type, the percentage of the total for each type, the number of plots in which nests were found, and the total number of nests with known fates in that crop type. A row for native sites is included at the bottom for comparison.

| **2020** | **# Plots** | **% Plots** | **w/Nest** | **# Nests** | **2021** | **# Plots** | **% Plots** | **w/Nest** | **# Nests** |
| --- | --- | --- | --- | --- | --- | --- | --- | --- | --- |
| Wheat | 14 | 56% | 12 | 31 |  | 17 | 63% | 12 | 20 |
| Su. Fallow | 4 | 16% | 3 | 7 |  | 8 | 30% | 3 | 4 |
| Lentil/Flax | 3 | 12% | 3 | 17 |  | 2 | 7% | 2 | 4 |
| Cover Crop | 2 | 8% | 2 | 5 |  | - | - | - | - |
| Pea | 1 | 4% | 1 | 3 |  | - | - | - | - |
| Canola | 1 | 4% | 1 | 5 |  | - | - | - | - |
| **Totals** | **25** | **100%** | **22** | **68** |  | **27** | **100%** | **17** | **28** |

| Native | **22** | **100%** | **18** | **71** |  | **28** | **100%** | **19** | **55** |
| --- | --- | --- | --- | --- | --- | --- | --- | --- | --- |

Table S2. Summary of numbers of nests used for each type of analysis. Shown are the total number of nests found in both site types (Nests Found), the total number with known fates used for nest survival analysis and to plot initiation dates (Known Fate), the number of successful nests out of those with known fates, from which apparent nest success was calculated (# Success), the number with known clutch sizes, from which mean clutch sizes were calculated (Known Clutch), the number that were found using behavioral search methods, from which a nest density index was calculated (Density), and the number of successful nests with known number fledged, for number of young fledged analysis (# Fledged).

|  | **Nests Found** | **Known Fate** | **# Success** | **Known Clutch** | **Density** | **# Fledged** |
| --- | --- | --- | --- | --- | --- | --- |
| **Crop** | 111 | 96 | 42 | 95 | 77 | 41 |
| **Native** | 129 | 126 | 46 | 125 | 91 | 46 |
| **Total** | **240** | **222** | **88** | **220** | **168** | **87** |

FIGURE CAPTIONS

Figure S1. Conceptualization of layout of line transect surveys used for thick-billed longspur abundance surveys in Valley County, Montana, 2020–21. The dashed lines represent the transect walked and the outer edge represents the 16-ha survey plot. Longspurs were recorded along with perpendicular distance and direction from the transect line.

Figure S2. Estimated probability of detecting a thick-billed longspur on a song meter recording relative to Julian day (top), daily minimum temperature (middle), and minutes past sunrise (bottom) in Valley County, Montana, during the month of April 2020–21. Results are from a multi-season occupancy analysis. Shaded regions depict 95% confidence intervals.

Figure S3. Effect of observer group (1 = high detection, 2 = low detection) on the distance detection function for thick-billed longspur surveys conducted in 2020 (top) and 2021 (bottom) in Valley County, Montana. Results are from open-population distance sampling analysis.
